# Supplementary material for: A Method for Intelligent Allocation of Diagnostic Testing by Leveraging Data from Commercial Wearable Devices: A Case Study on COVID-19
Source: Res Sq. 2022 Apr 1:rs.3.rs-1490524. Preprint. [Version 1] doi: 10.21203/rs.3.rs-1490524/v1 (PMC8978951; doi:10.21203/rs.3.rs-1490524/v1)
Supplement: Supplement 1 [file a0dd3f6fe9a58360b18ab15b.pdf]

Consent Form to Participate in Research for CovIdentify

This study is being conducted by Dr. Jessilyn Dunn, PhD and Dr. Ryan Shaw, RN, PhD of the Duke University Department of Biomedical Engineering and Schools of Nursing and Medicine.

Key Information

"CovIdentify" is a new study to help researchers detect COVID-19 infection. Anyone who has a computer or smart phone, and is over the age of 18 can participate. If you have a smart watch or wearable device, you can choose to contribute data from the device (Not required to participate). Participation is completely your choice. We are asking people all over the world to join our COVID-19 study. We will collect daily or weekly surveys that ask how you have been feeling and about social distancing. The information we gather will be kept in databases on password-protected servers.

WHY IS THIS STUDY BEING DONE?

We have learned how to use smart watch data to detect early signs of illness. We are doing this study to help us learn more about COVID-19.

WHAT WILL I BE ASKED TO DO?

You will be asked to provide informed consent, complete a baseline survey about yourself, and then brief daily or weekly surveys on social distancing and testing. If you have a wearable device, you will be asked to contribute data such as heart rate and activity. All survey questions can be completed on a smartphone, tablet, or laptop/computer.

HOW LONG WILL I BE IN THE STUDY?

This study will last up to 12 months. You may decline to answer any questions or the entire survey or withdraw from the study at any time.

Risks

If a survey question makes you feel uncomfortable, you may choose not to answer it. Every effort will be made to keep your information confidential. The information we gather will be kept in databases on password-protected servers. When we report what we learn from this study, no individual study participant will be identifiable. Data from this study may be used for future research, but in a way that no individual participant will be identifiable.
Benefits

Participation in this study may be of no benefit to you. We hope information learned from this study will help with COVID-19 research. We will not provide you with information about your chances of having or getting the virus.

WHAT ABOUT COMPENSATION?

Participants in this study can purchase a Fitbit or Garmin device at a discount while supplies last. Depending on your risk of contracting COVID-19, you may be eligible to receive a smart watch as a part of this study. Please email covidentify@duke.edu to learn more.

WILL MY INFORMATION BE KEPT CONFIDENTIAL?

Data will be stored on Duke's protected network and approved cloud services and is only available to approved research personnel. Only the researchers on this study will have access to your information. Your survey responses and device data will be given a study ID number. The key that connects your study ID number with your identifying information, for example, your name or email address, will be stored separate from your survey responses and device data. We will not analyze data on an individual level (i.e., for each participant). The data may be used in future analyses by the study investigators and in aggregate with other investigators to discover needs related to COVID-19.

It is important that you review and understand the terms and conditions for using your wearable device. You must agree to their terms of service and download the respective companion apps.

For Apple users, see https://www.apple.com/privacy/
For Fitbit users, see https://www.fitbit.com/legal/privacy-policy
For Garmin users, see https://www.garmin.com/en-US/privacy/connect/

For Fitbit, Garmin, and Android users, we will ask you to provide an email address and cell phone number associated with these devices which we will store securely.

We do not collect GPS data or real-time location data. You have the option of telling us your address, and this information will be used by Duke researchers to understand the spread of COVID-19 over time, and how location corresponds to risk of infection.

VOLUNTARY NATURE OF PARTICIPATION

Participation in this study is voluntary. You can choose not to participate at any point and may withdraw at any time. Please opt-out from communications or delete the CovIdentify app and notify the study staff if you wish to withdraw. After withdrawal, Covidentify will no longer obtain further information from you, but still has permission to use the information collected previously.

CONTACT FOR FUTURE STUDIES

The information we obtain in this study may be used again in future research related to Covidentify. Please check the box if you wish to be re-contacted for future research opportunities.
{[consent7] radio}

◯ {1} Please re-contact me about future research opportunities such as the impact of the pandemic on autoimmune disease, risk factors such as diabetes or hypertension, or the use of wearable devices.

WHOM DO I CALL IF I HAVE QUESTIONS OR PROBLEMS?

For questions about the study, please contact the study team at covidentify@duke.edu or Dr. Jessilyn Dunn at 919-668-9798 during regular business hours. For questions about your rights as a participant in this research study, contact the Duke Campus IRB at 919-684-3030 or campusirb@duke.edu.

PARTICIPATION WHILE TRAVELING IN THE EUROPEAN ECONOMIC AREA (EEA: AN EXTENSION OF THE EUROPEAN UNION) UNDER THE GENERAL DATA PROTECTION REGULATION (GDPR) AND STATEMENT OF CONSENT

If you live in or are traveling in specific regions of Europe (the European Economic Area, or EEA), you are subject to additional data protection laws called "General Data Protection Regulations" (GDPR). To participate in our study, you must check the box below. By checking the box, you give us permission to collect and process your sensitive personal data while you are in the EEA, as well as transfer your data to the United States.

If you are in or travel to the EEA during this study, we are required to inform you that sensitive personal data about you, including racial or ethnic origin, genetic or biometric information, and health information, will be collected.

{[consent11] radio}

◯ {1} I have read the consent form and I wish to participate in the study AND I consent to the collection and transfer of my personal data, including my sensitive personal data, if I am traveling to or living in the EEA during the course of this study

---

If you agree to take part in the study, please sign and date below.
{Branching logic (show if): [consent11] = '1'}

Date of Birth
{[dob] text date_mdy}
{Branching logic (show if): [consent11] = '1'}

____

Age:
{[age] calc}
{Branching logic (show if): [consent11] = '1'}

____

Please note that based on your selection, you will not be enrolled in this study.
{Branching logic (show if): [age] < 18 and [dob]''}

First Name
{[fname] text}
{Branching logic (show if): [consent11] = '1' and [age]>=18}

____

Last Name
{[lname] text}
{Branching logic (show if): [consent11] = '1' and [age]>=18}

____

E-mail Address
{[email_consent] text email}
{Branching logic (show if): ([consent11] = '1' or [consent7] ='1') and [age]>=18}

____

Signature
{[signature] file signature}
{Branching logic (show if): [consent11] = '1' and [age]>=18}

____
(Sign using your mouse on your computer or finger on your cell phone)

Consent Date
{[new_consent] text date_mdy}

____

Consent Version Date
{[version_dte] text date_mdy}

____

Consent Expiration Date
{[exp_dte] text date_mdy}

____

Enrollment

Thanks for giving us some background.

These questions are essential to help our research team identify symptoms of COVID-19 infection and validate results.	
Do you actively use a wearable device (e.g. Fitbit, Garmin, or Applewatch)?<br>{[own_wearable] radio}	◯ {1} Yes ◯ {0} No
Which wearable device brands do you use most frequently?<br>{[wearable_device] radio}<br>{Branching logic (show if): [consent_arm_1][consent11] = '1' and [own_wearable] = '1'}	◯ {1} Fitbit<br>◯ {2} Apple Watch<br>◯ {3} Garmin<br>◯ {555} Other<br>(Check all that apply)
Specify Other<br>{[oth_device] text}<br>{Branching logic (show if): [wearable_device] = '555'}	______________________________

COVID-19 Testing	
Have you ever been tested for COVID-19?<br>{[tested_covid] radio}<br>{Branching logic (show if): [consent_arm_1][consent11] = '1'}	◯ {1} Yes ◯ {0} No<br>◯ {999} I don't know
How many times have you tested positive for COVID-19?<br><br>{[number_positive_covid_test] radio}<br>{Branching logic (show if): [tested_covid] = '1'}	◯ {1} 0 (never tested positive)<br>◯ {2} 1 ◯ {3} 2 or more times
What were the dates of positive COVID testing (e.g. day of nasal swab)? List up to three most recent test dates.<br>{Branching logic (show if): [number_positive_covid_test] = '2' or [number_positive_covid_test] = '3'}	
What was the date of the first COVID-19 test?<br>{[covid_positive_date_1] text date_mdy}<br>{Branching logic (show if):<br>[number_positive_covid_test] = '2' or<br>[number_positive_covid_test] = '3'}	______________________________
What type of test was it?<br>{[covid_positive_type_1] radio}<br>{Branching logic (show if):<br>[number_positive_covid_test] = '2' or<br>[number_positive_covid_test] = '3'}	◯ {1} Antigen (rapid 15 minute test)<br>◯ {2} Diagnostic (PCR)<br>◯ {3} Antibody (via blood draw)<br>◯ {4} Don't know
What was the date of the second COVID-19 test if you had one? If not, please leave it blank.<br>{[covid_positive_date_2] text date_mdy}<br>{Branching logic (show if):<br>[number_positive_covid_test] = '2' or<br>[number_positive_covid_test] = '3'}	______________________________

What type of test was it?<br>{[covid_positive_type_2] radio}<br>{Branching logic (show if):<br>[number_positive_covid_test] = '2' or<br>[number_positive_covid_test] = '3'}	◯ {1} Antigen (rapid 15 minute test)<br>◯ {2} Diagnostic (PCR)<br>◯ {3} Antibody (via blood draw)
What was the date of the third COVID-19 test if you had one? If not, please leave it blank.<br>{[covid_positive_date_3] text date_mdy}<br>{Branching logic (show if):<br>[number_positive_covid_test] = '2' or<br>[number_positive_covid_test] = '3'}	________________________
What type of test was it?<br>{[covid_positive_type_3] radio}<br>{Branching logic (show if):<br>[number_positive_covid_test] = '2' or<br>[number_positive_covid_test] = '3'}	◯ {1} Antigen (rapid 15 minute test)<br>◯ {2} Diagnostic (PCR)<br>◯ {3} Antibody (via blood draw)

COVID-19 Vaccine

Have you received the COVID-19 vaccine?<br>{[covid_shot_enrollment] radio}	◯ {1} Yes ◯ {2} No
Have you received the first dose of the COVID-19 vaccine?<br>{[covid_first_shot] radio}<br>{Branching logic (show if): [consent_arm_1][consent11] = '1' and [covid_shot_enrollment] = '1'}	◯ {1} Yes ◯ {0} No
What was the date of the first dose?<br>{[covid_first_shot_date] text date_mdy}<br>{Branching logic (show if): [covid_first_shot] = '1'}	________________________
Do you know the brand?<br>{[covid_first_shot_brand] radio}<br>{Branching logic (show if): [covid_first_shot] = '1'}	◯ {1} Pfizer<br>◯ {2} Moderna<br>◯ {12} Johnson & Johnson<br>◯ {3} Other<br>◯ {11} I don't know

Did you have any symptoms that you believe were caused by the first dose?

	{0} None	{1} Mild	{2} Moderate	{3} Severe	{4} Very Severe
Fever<br>{[fever_first_shot] radio}<br>{Branching logic (show if):<br>[covid_first_shot] = '1'}	◯	◯	◯	◯	◯
Generally feeling ill (Malaise)<br>{[malaise_first_shot] radio}<br>{Branching logic (show if):<br>[covid_first_shot] = '1'}	◯	◯	◯	◯	◯

Cough {[cough1_first_shot] radio} {Branching logic (show if): [covid_first_shot] = '1'}	○	○	○	○	○
Sneezing {[sneezing_first_shot] radio} {Branching logic (show if): [covid_first_shot] = '1'}	○	○	○	○	○
Shortness of breath/difficulty breathing {[shortness1_first_shot] radio} {Branching logic (show if): [covid_first_shot] = '1'}	○	○	○	○	○
Muscle Aches {[muscle_aches_first_shot] radio} {Branching logic (show if): [covid_first_shot] = '1'}	○	○	○	○	○
Runny/stuffy nose {[runny_nose_first_shot] radio} {Branching logic (show if): [covid_first_shot] = '1'}	○	○	○	○	○
Throat discomfort (sore/scratchy throat) {[sore_throat1_first_shot] radio} {Branching logic (show if): [covid_first_shot] = '1'}	○	○	○	○	○
Nausea or vomiting {[nausea1_first_shot] radio} {Branching logic (show if): [covid_first_shot] = '1'}	○	○	○	○	○
Headache {[headache1_first_shot] radio} {Branching logic (show if): [covid_first_shot] = '1'}	○	○	○	○	○
Abdominal pain {[abd_pain1_first_shot] radio} {Branching logic (show if): [covid_first_shot] = '1'}	○	○	○	○	○
Diarrhea {[diarrhea1_first_shot] radio} {Branching logic (show if): [covid_first_shot] = '1'}	○	○	○	○	○
Lost or changed of sense of taste {[loss_taste_first_shot] radio} {Branching logic (show if): [covid_first_shot] = '1'}	○	○	○	○	○

Lost or changed of sense of smell {[loss_smell_first_shot] radio} {Branching logic (show if): [covid_first_shot] = '1'}	◯	◯	◯	◯	◯
Arm Soreness {[arm_sore_first_shot] radio} {Branching logic (show if): [covid_first_shot] = '1'}	◯	◯	◯	◯	◯
Other {[oth1_first_shot] radio} {Branching logic (show if): [covid_first_shot] = '1'}	◯	◯	◯	◯	◯

Have you received the second dose of the COVID-19 vaccine?
{[covid_second_shot] radio}
{Branching logic (show if): [covid_shot_enrollment] = '1'}

◯ {1} Yes ◯ {0} No

Do you know the brand?
{[covid_second_shot_brand] radio}
{Branching logic (show if): [covid_second_shot] = '1'}

◯ {1} Pfizer
◯ {2} Moderna
◯ {12} Johnson & Johnson
◯ {3} Other
◯ {11} I don't know

Did you have any symptoms that you believe were caused by the second dose?

	{0} None	{1} Mild	{2} Moderate	{3} Severe	{4} Very Severe
Fever {[fever_second_shot] radio} {Branching logic (show if): [covid_second_shot] = '1'}	◯	◯	◯	◯	◯
Generally feeling ill (Malaise) {[malaise_second_shot] radio} {Branching logic (show if): [covid_second_shot] = '1'}	◯	◯	◯	◯	◯
Cough {[cough1_second_shot] radio} {Branching logic (show if): [covid_second_shot] = '1'}	◯	◯	◯	◯	◯
Sneezing {[sneezing_second_shot] radio} {Branching logic (show if): [covid_second_shot] = '1'}	◯	◯	◯	◯	◯
Shortness of Breath/Difficulty breathing {[shortness1_second_shot] radio} {Branching logic (show if): [covid_second_shot] = '1'}	◯	◯	◯	◯	◯

Muscle Aches {[muscle_aches_second_shot] radio} {Branching logic (show if): [covid_second_shot] = '1'}	○	○	○	○	○
Runny/stuffy nose {[runny_nose_second_shot] radio} {Branching logic (show if): [covid_second_shot] = '1'}	○	○	○	○	○
Throat discomfort (sore/scratchy throat) {[sore_throat1_second_shot] radio} {Branching logic (show if): [covid_second_shot] = '1'}	○	○	○	○	○
Nausea or vomiting {[nausea1_second_shot] radio} {Branching logic (show if): [covid_second_shot] = '1'}	○	○	○	○	○
Headache {[headache1_second_shot] radio} {Branching logic (show if): [covid_second_shot] = '1'}	○	○	○	○	○
Abdominal pain {[abd_pain1_second_shot] radio} {Branching logic (show if): [covid_second_shot] = '1'}	○	○	○	○	○
Diarrhea {[diarrhea1_second_shot] radio} {Branching logic (show if): [covid_second_shot] = '1'}	○	○	○	○	○
Lost or changed of sense of taste {[loss_taste_second_shot] radio} {Branching logic (show if): [covid_second_shot] = '1'}	○	○	○	○	○
Lost or changed of sense of smell {[loss_smell_second_shot] radio} {Branching logic (show if): [covid_second_shot] = '1'}	○	○	○	○	○
Arm Soreness {[arm_sore_second_shot] radio} {Branching logic (show if): [covid_second_shot] = '1'}	○	○	○	○	○
Other {[oth1_second_shot] radio} {Branching logic (show if): [covid_second_shot] = '1'}	○	○	○	○	○

What was the date of the second dose?
{[covid_second_shot_date] text date_mdy}
{Branching logic (show if): [covid_second_shot] = '1'}

____

Have you received the third dose (first booster) of the COVID-19 vaccine?
{[covid_third_shot] radio}
{Branching logic (show if): [covid_shot_enrollment] = '1'}

◯ {1} Yes ◯ {2} No

What was the date of the third shot?
{[covid_third_shot_date] text date_mdy}
{Branching logic (show if): [covid_third_shot] = '1'}

____

Do you know the brand?
{[covid_third_shot_brand] radio}
{Branching logic (show if): [covid_third_shot] = '1'}

◯ {1} Pfizer
◯ {2} Moderna
◯ {12} Johnson & Johnson
◯ {3} Other
◯ {11} I don't know

Did you have any symptoms that you believe were caused by the third dose?

	{0} None	{1} Mild	{2} Moderate	{3} Severe	{4} Very Severe
Fever {[fever_third_shot] radio} {Branching logic (show if): [covid_third_shot] = '1'}	◯	◯	◯	◯	◯
Generally Feeling ill (Malaise) {[malaise_third_shot] radio} {Branching logic (show if): [covid_third_shot] = '1'}	◯	◯	◯	◯	◯
Cough {[cough1_third_shot] radio} {Branching logic (show if): [covid_third_shot] = '1'}	◯	◯	◯	◯	◯
Sneezing {[sneezing_third_shot] radio} {Branching logic (show if): [covid_third_shot] = '1'}	◯	◯	◯	◯	◯
Shortness of Breath/Difficulty breathing {[shortness1_third_shot] radio} {Branching logic (show if): [covid_third_shot] = '1'}	◯	◯	◯	◯	◯
Muscle Aches {[muscle_aches_third_shot] radio} {Branching logic (show if): [covid_third_shot] = '1'}	◯	◯	◯	◯	◯
Runny/stuffy noses {[runny_nose_third_shot] radio} {Branching logic (show if): [covid_third_shot] = '1'}	◯	◯	◯	◯	◯

Throat discomfort (sore/scratchy throat) {[sore_throat1_third_shot] radio} {Branching logic (show if): [covid_third_shot] = '1'}	◯	◯	◯	◯	◯
Nausea or vomiting {[nausea1_third_shot] radio} {Branching logic (show if): [covid_third_shot] = '1'}	◯	◯	◯	◯	◯
Headache {[headache1_third_shot] radio} {Branching logic (show if): [covid_third_shot] = '1'}	◯	◯	◯	◯	◯
Abdominal Pain {[abd_pain1_third_shot] radio} {Branching logic (show if): [covid_third_shot] = '1'}	◯	◯	◯	◯	◯
Diarrhea {[diarrhea1_third_shot] radio} {Branching logic (show if): [covid_third_shot] = '1'}	◯	◯	◯	◯	◯
Lost or changed of sense of smell {[loss_taste_third_shot] radio} {Branching logic (show if): [covid_third_shot] = '1'}	◯	◯	◯	◯	◯
Arm Soreness {[arm_sore_third_shot] radio} {Branching logic (show if): [covid_third_shot] = '1'}	◯	◯	◯	◯	◯
Other {[oth1_third_shot] radio} {Branching logic (show if): [covid_third_shot] = '1'}	◯	◯	◯	◯	◯

Have you received the fourth dose (second booster) of the COVID-19 vaccine?
{[covid_fourth_shot] radio}
{Branching logic (show if): [covid_shot_enrollment] = '1'}

◯ {1} Yes ◯ {2} No

What was the date of the fourth shot?
{[covid_fourth_shot_date] text date_mdy}
{Branching logic (show if): [covid_fourth_shot] = '1'}

____

Do you know the brand?
{[covid_fourth_shot_brand] radio}
{Branching logic (show if): [covid_fourth_shot] = '1'}

◯ {1} Pfizer
◯ {2} Moderna
◯ {12} Johnson & Johnson
◯ {3} Other
◯ {11} I don't know

Did you have any symptoms that you believe were caused by the fourth dose?

	{0} None	{1} Mild	{2} Moderate	{3} Severe	{4} Very Severe
Fever {[fever_fourth_shot] radio} {Branching logic (show if): [covid_fourth_shot] = '1'}	○	○	○	○	○
Generally Feeling ill (Malaise) {[malaise_fourth_shot] radio} {Branching logic (show if): [covid_fourth_shot] = '1'}	○	○	○	○	○
Cough {[cough_fourth_shot] radio} {Branching logic (show if): [covid_fourth_shot] = '1'}	○	○	○	○	○
Sneezing {[sneezing_fourth_shot] radio} {Branching logic (show if): [covid_fourth_shot] = '1'}	○	○	○	○	○
Shortness of Breath/Difficulty breathing {[shortness1_fourth_shot] radio} {Branching logic (show if): [covid_fourth_shot] = '1'}	○	○	○	○	○
Muscle Aches {[muscle_aches_fourth_shot] radio} {Branching logic (show if): [covid_fourth_shot] = '1'}	○	○	○	○	○
Runny/stuffy noses {[runny_nose_fourth_shot] radio} {Branching logic (show if): [covid_fourth_shot] = '1'}	○	○	○	○	○
Throat discomfort (sore/scratchy throat) {[sore_throat1_fourth_shot] radio} {Branching logic (show if): [covid_fourth_shot] = '1'}	○	○	○	○	○
Nausea or vomiting {[nausea1_fourth_shot] radio} {Branching logic (show if): [covid_fourth_shot] = '1'}	○	○	○	○	○
Headache {[headache1_fourth_shot] radio} {Branching logic (show if): [covid_fourth_shot] = '1'}	○	○	○	○	○
Abdominal Pain {[abd_pain1_fourth_shot] radio} {Branching logic (show if): [covid_fourth_shot] = '1'}	○	○	○	○	○

Diarrhea {[diarrhea1_fourth_shot] radio} {Branching logic (show if): [covid_fourth_shot] = '1'}	◯	◯	◯	◯	◯
Lost or changed of sense of smell {[loss_taste_fourth_shot] radio} {Branching logic (show if): [covid_fourth_shot] = '1'}	◯	◯	◯	◯	◯
Arm Soreness {[arm_sore_fourth_shot] radio} {Branching logic (show if): [covid_fourth_shot] = '1'}	◯	◯	◯	◯	◯
Other {[oth1_fourth_shot] radio} {Branching logic (show if): [covid_fourth_shot] = '1'}	◯	◯	◯	◯	◯

Flu Testing

Did you have an influenza-like illness (flu) in the past year? {[had_flu] radio} {Branching logic (show if): [consent_arm_1][consent11] = '1'}	◯ {1} Yes ◯ {0} No ◯ {999} Unknown
What was the first day you felt sick (approximately)? {[had_flu_date] text date_mdy} {Branching logic (show if): [had_flu] = '1'}	______________________
Did you get the flu shot? {[flu_shot] radio} {Branching logic (show if): [consent_arm_1][consent11] = '1'}	◯ {1} Yes ◯ {0} No ◯ {999} Maybe
If you remember, when did you get the flu shot? {[flu_shot_date] text date_mdy} {Branching logic (show if): [consent_arm_1][consent11] = '1' and [flu_shot] = '1'}	______________________

Demographics:

Biological sex at birth {[sex] dropdown} {Branching logic (show if): [consent_arm_1][consent11] = '1'}	◯ {1} Male ◯ {0} Female ◯ {777} Unknown ◯ {555} Other
Specify Other {[oth_sex] text} {Branching logic (show if): [sex] = '555'}	______________________
Gender Identity {[gender_identity] dropdown} {Branching logic (show if): [consent_arm_1][consent11] = '1'}	◯ {0} Female ◯ {1} Male ◯ {2} Non-binary/third gender ◯ {3} Prefer to self-describe ◯ {4} Prefer not to say

Please describe<br>{[desc_gender] text}<br>{Branching logic (show if): [gender_identity] = '3'}	________________________
Race<br>{[race] checkbox}<br>{Branching logic (show if): [consent_arm_1][consent11] = '1'}	☐ {0} White<br>☐ {1} Black or African American<br>☐ {2} Asian<br>☐ {3} Native American or Alaska Native<br>☐ {4} Native Hawaiian or Pacific Islander<br>☐ {999} Other<br>☐ {5} Prefer not to say/Unknown<br>(Check all that apply)
Ethnicity<br>{[ethnicity] radio}<br>{Branching logic (show if): [consent_arm_1][consent11] = '1'}	◯ {0} Not Hispanic or Latino<br>◯ {1} Hispanic or Latino<br>◯ {999} Unknown or Not Reported

Country
{[country] dropdown}
{Branching logic (show if): [consent_arm_1][consent11] = '1'}

◯ {186} United States of America (USA)
◯ {1} Afghanistan
◯ {2} Albania
◯ {3} Algeria
◯ {4} Andorra
◯ {5} Angola
◯ {6} Antigua and Barbuda
◯ {7} Argentina
◯ {8} Armenia
◯ {9} Australia
◯ {10} Austria
◯ {11} Azerbaijan
◯ {12} Bahamas
◯ {13} Bahrain
◯ {14} Bangladesh
◯ {15} Barbados
◯ {16} Belarus
◯ {17} Belgium
◯ {18} Belize
◯ {19} Benin
◯ {20} Bhutan
◯ {21} Bolivia
◯ {22} Bosnia and Herzegovina
◯ {23} Botswana
◯ {24} Brazil
◯ {25} Brunei
◯ {26} Bulgaria
◯ {27} Burkina Faso
◯ {28} Burundi
◯ {29} Cabo Verde
◯ {30} Cambodia
◯ {31} Cameroon
◯ {32} Canada
◯ {33} Central African Republic (CAR)
◯ {34} Chad
◯ {35} Chile
◯ {36} China
◯ {37} Colombia
◯ {38} Comoros
◯ {196} Congo, Democratic Republic of the Congo
◯ {39} Costa Rica
◯ {40} Cote d'Ivoire
◯ {41} Croatia
◯ {42} Cuba
◯ {43} Cyprus
◯ {44} Czechia
◯ {45} Denmark
◯ {46} Djibouti
◯ {47} Dominica
◯ {48} Dominican Republic
◯ {49} Ecuador
◯ {50} Egypt
◯ {51} El Salvador
◯ {52} Equatorial Guinea
◯ {53} Eritrea
◯ {54} Estonia
◯ {55} Eswatini (formerly Swaziland)
◯ {56} Ethiopia
◯ {57} Fiji
◯ {58} Finland
◯ {59} France
◯ {60} Gabon
◯ {61} Gambia
◯ {62} Georgia
◯ {63} Germany
◯ {64} Ghana
◯ {65} Greece
◯ {66} Grenada
◯ {67} Guatemala

◯ {68} Guinea
◯ {69} Guinea-Bissau
◯ {70} Guyana
◯ {71} Haiti
◯ {72} Honduras
◯ {73} Hungary
◯ {74} Iceland
◯ {75} India
◯ {76} Indonesia
◯ {77} Iran
◯ {78} Iraq
◯ {79} Ireland
◯ {80} Israel
◯ {81} Italy
◯ {82} Jamaica
◯ {83} Japan
◯ {84} Jordan
◯ {85} Kazakhstan
◯ {86} Kenya
◯ {87} Kiribati
◯ {88} Kosovo
◯ {89} Kuwait
◯ {90} Kyrgyzstan
◯ {91} Laos
◯ {92} Latvia
◯ {93} Lebanon
◯ {94} Lesotho
◯ {95} Liberia
◯ {96} Libya
◯ {97} Liechtenstein
◯ {98} Lithuania
◯ {99} Luxembourg
◯ {100} Madagascar
◯ {101} Malawi
◯ {102} Malaysia
◯ {103} Maldives
◯ {104} Mali
◯ {105} Malta
◯ {106} Marshall Islands
◯ {107} Mauritania
◯ {108} Mauritius
◯ {109} Mexico
◯ {110} Micronesia
◯ {111} Moldova
◯ {112} Monaco
◯ {113} Mongolia
◯ {114} Montenegro
◯ {115} Morocco
◯ {116} Mozambique
◯ {117} Myanmar (formerly Burma)
◯ {118} Namibia
◯ {119} Nauru
◯ {120} Nepal
◯ {121} Netherlands
◯ {122} New Zealand
◯ {123} Nicaragua
◯ {124} Niger
◯ {125} Nigeria
◯ {126} North Korea
◯ {127} North Macedonia (formerly Macedonia)
◯ {128} Norway
◯ {129} Oman
◯ {130} Pakistan
◯ {131} Palau
◯ {132} Palestine
◯ {133} Panama
◯ {134} Papua New Guinea
◯ {135} Paraguay
◯ {136} Peru
◯ {137} Philippines
◯ {138} Poland

◯ {139} Portugal
◯ {140} Qatar
◯ {141} Romania
◯ {142} Russia
◯ {143} Rwanda
◯ {144} Saint Kitts and Nevis
◯ {145} Saint Lucia
◯ {146} Saint Vincent and the Grenadines
◯ {147} Samoa
◯ {148} San Marino
◯ {149} Sao Tome and Principe
◯ {150} Saudi Arabia
◯ {151} Senegal
◯ {152} Serbia
◯ {153} Seychelles
◯ {154} Sierra Leone
◯ {155} Singapore
◯ {156} Slovakia
◯ {157} Slovenia
◯ {158} Solomon Islands
◯ {159} Somalia
◯ {160} South Africa
◯ {161} South Korea
◯ {162} South Sudan
◯ {163} Spain
◯ {164} Sri Lanka
◯ {165} Sudan
◯ {166} Suriname
◯ {167} Sweden
◯ {168} Switzerland
◯ {169} Syria
◯ {170} Taiwan
◯ {171} Tajikistan
◯ {172} Tanzania
◯ {173} Thailand
◯ {174} Timor-Leste
◯ {175} Togo
◯ {176} Tonga
◯ {177} Trinidad and Tobago
◯ {178} Tunisia
◯ {179} Turkey
◯ {180} Turkmenistan
◯ {181} Tuvalu
◯ {182} Uganda
◯ {183} Ukraine
◯ {184} United Arab Emirates (UAE)
◯ {185} United Kingdom (UK)
◯ {187} Uruguay
◯ {188} Uzbekistan
◯ {189} Vanuatu
◯ {190} Vatican City (Holy See)
◯ {191} Venezuela
◯ {192} Vietnam
◯ {193} Yemen
◯ {194} Zambia
◯ {195} Zimbabwe
◯ {555} Other

Specify Other
{[oth_country] text}
{Branching logic (show if): [country] = '555'}

____

Zip Code
{[zip] text zipcode}
{Branching logic (show if): [country]='186'}

____

Postal Code<br>{[postal_code] text}<br>{Branching logic (show if): [country] != '186'}	______________________
What kind of phone do you have?<br>{[phone_type] radio}<br>{Branching logic (show if): [consent_arm_1][consent11] = '1'}	◯ {1} iPhone ◯ {2} Android<br>◯ {555} Other
Specify Other<br>{[oth_phone] text}<br>{Branching logic (show if): [phone_type] = '555'}	______________________
What is your profession or occupation?<br>{[profession] dropdown autocomplete}<br>{Branching logic (show if): [consent_arm_1][consent11] = '1'}	◯ {1} Management Occupations<br>◯ {2} Business and Financial Operations Occupations<br>◯ {3} Computer and Mathematical Occupations<br>◯ {4} Architecture and Engineering Occupations<br>◯ {5} Life, Physical, and Social Science Occupations<br>◯ {6} Community and Social Service Occupations<br>◯ {7} Legal Occupations<br>◯ {8} Educational Instruction and Library Occupations<br>◯ {9} Arts, Design, Entertainment, Sports, and Media Occupations<br>◯ {10} Healthcare Practitioners and Technical Occupations<br>◯ {11} Healthcare Support Occupations<br>◯ {12} Protective Service Occupations<br>◯ {13} Food Preparation and Serving Related Occupations<br>◯ {14} Building and Grounds Cleaning and Maintenance Occupations<br>◯ {15} Personal Care and Service Occupations<br>◯ {16} Sales and Related Occupations<br>◯ {17} Office and Administrative Support Occupations<br>◯ {18} Farming, Fishing, and Forestry Occupations<br>◯ {19} Construction and Extraction Occupations<br>◯ {20} Installation, Maintenance, and Repair Occupations<br>◯ {21} Production Occupations<br>◯ {22} Transportation and Material Moving Occupations<br>◯ {23} Military Specific Occupations
Type of Residence<br>{[typ_residence] dropdown}<br>{Branching logic (show if): [consent_arm_1][consent11] = '1'}	◯ {1} Private residence<br>◯ {9} Apartment<br>◯ {2} School dormitory<br>◯ {3} Nursing home/LTC facility<br>◯ {4} Correctional setting<br>◯ {5} Military base<br>◯ {6} Shelter<br>◯ {7} Homeless<br>◯ {8} Unknown<br>◯ {555} Other
Specify Other<br>{[oth_residence] text}<br>{Branching logic (show if): [typ_residence] = '555'}	______________________
Have you participated in CovIdentify previously?<br>{[prev_cov_proj] yesno}<br>{Branching logic (show if): [consent_arm_1][consent11] = '1'}	◯ Yes ◯ No

Do you smoke or vape?<br>{[smoke] radio}<br>{Branching logic (show if): [consent_arm_1][consent11] = '1'}	◯ {1} Smoke ◯ {2} Vape<br>◯ {3} Neither
How many packs per day?<br>{[packs_day] text}<br>{Branching logic (show if): [smoke] = '1'}	____________________
How many cartridges per day?<br>{[cartridges_day] text}<br>{Branching logic (show if): [smoke] = '2'}	____________________
Please select any of the following medical conditions you have:<br>{[med_conditions] checkbox}<br>{Branching logic (show if): [consent_arm_1][consent11] = '1'}	☐ {1} Pregnancy<br>☐ {2} Diabetes<br>☐ {3} Cardiac Disease<br>☐ {4} Chronic pulmonary disease<br>☐ {5} Chronic kidney disease<br>☐ {6} Chronic liver disease<br>☐ {7} Immunocompromised<br>☐ {8} Hypertension<br>☐ {9} Asthma<br>☐ {10} Seasonal allergies<br>☐ {11} Lupus<br>☐ {12} Rheumatoid arthritis<br>☐ {13} Other autoimmune condition<br>☐ {555} Other<br>☐ {999} Unknown<br>(Check all that apply)
# of weeks pregnant<br>{[weeks_preg] text float}<br>{Branching logic (show if): [med_conditions(1)] = '1'}	____________________<br>(Weeks)
Are you participating in the Duke Lupus Registry?<br>{[lupus_registry] radio}<br>{Branching logic (show if): [med_conditions(11)]='1'}	◯ {1} Yes ◯ {0} No<br>◯ {999} Unknown
Are you a participant in the Duke Snowball Study?<br>{[snowball_study] yesno}<br>{Branching logic (show if): [consent_arm_1][consent11] = '1'}	◯ Yes<br>◯ No
Specify Other<br>{[oth_med_cond] text}<br>{Branching logic (show if): [med_conditions(555)] = '1'}	____________________
Blood type<br>{[blood_type] radio}<br>{Branching logic (show if): [consent_arm_1][consent11] = '1'}	◯ {1} A-<br>◯ {2} A+<br>◯ {3} B-<br>◯ {4} B+<br>◯ {5} AB-<br>◯ {6} AB+<br>◯ {7} O-<br>◯ {8} O+<br>◯ {999} Don't Know

Are you on any medications?
{[medications] yesno}
{Branching logic (show if): [consent_arm_1][consent11] = '1'}

◯ Yes ◯ No

Please specify medication(s) and dose:
{[spef_meds] textarea}
{Branching logic (show if): [medications] = '1'}

How did you find out about CovIdentify?
{[survey_discovery] checkbox}
{Branching logic (show if): [consent_arm_1][consent11] = '1'}

☐ {1} Facebook/Instagram
☐ {2} Email
☐ {3} Flyer/Card at Testing Facility
☐ {4} Word of Mouth
☐ {5} Other

Thank you for completing the enrollment survey! A link for the daily survey will be sent immediately after the completion of this survey with some follow-up questions. We greatly appreciate your time and support in helping us better understand COVID-19.

FOR CovIdentify Team ONLY

Previous CovIdentify Project
{[prev_cov_project] dropdown}

◯ {9148} 9148 - CovIdentify
◯ {9363} 9363 - CovIdentify - Emails

Previous Record ID
{[previous_record_id] text}

____

Secondary ID
{[secondary_id] text}

____

Touched Record
{[touch_rec] yesno}

◯ Yes ◯ No

Opt-Out (If greater that 1, then participant has opted out)
{[optout_sum] calc}

____

We apologize for any inconvenience, but at this time you are not eligible to participate in this study.

Daily Questionnaire: CovIdentify

Have you been notified of potential exposure to COVID-19 in the past 24 hours?<br><br>{[exposed_covid] radio}	◯ {1} Yes ◯ {2} No
Did you feel sick in the past 24 hours?<br>{[sick_today] radio}	◯ {1} Yes ◯ {0} No

Do you have any of the following symptoms?

	{0} None	{1} Mild	{2} Moderate	{3} Severe	{4} Very Severe
Fever<br>{[fever] radio}<br>{Branching logic (show if):<br>[sick_today] = '1' or [sick_today]<br>= '2'}	◯	◯	◯	◯	◯
Generally feeling ill (Malaise)<br>{[malaise] radio}<br>{Branching logic (show if):<br>[sick_today] = '1' or [sick_today]<br>= '2'}	◯	◯	◯	◯	◯
Cough<br>{[cough1] radio}<br>{Branching logic (show if):<br>[sick_today] = '1' or [sick_today]<br>= '2'}	◯	◯	◯	◯	◯
Sneezing<br>{[sneezing] radio}<br>{Branching logic (show if):<br>[sick_today] = '1' or [sick_today]<br>= '2'}	◯	◯	◯	◯	◯
Shortness of breath/difficulty breathing<br>{[shortness1] radio}<br>{Branching logic (show if):<br>[sick_today] = '1' or [sick_today]<br>= '2'}	◯	◯	◯	◯	◯
Muscle Aches<br>{[muscle_aches1] radio}<br>{Branching logic (show if):<br>[sick_today] = '1' or [sick_today]<br>= '2'}	◯	◯	◯	◯	◯
Runny/stuffy nose<br>{[runny_nose] radio}<br>{Branching logic (show if):<br>[sick_today] = '1' or [sick_today]<br>= '2'}	◯	◯	◯	◯	◯
Throat discomfort (sore/scratchy throat)<br>{[sore_throat1] radio}<br>{Branching logic (show if):<br>[sick_today] = '1' or [sick_today]<br>= '2'}	◯	◯	◯	◯	◯

Nausea or vomiting {[nausea1] radio} {Branching logic (show if): [sick_today] = '1' or [sick_today] = '2'}	◯	◯	◯	◯	◯
Headache {[headache1] radio} {Branching logic (show if): [sick_today] = '1' or [sick_today] = '2'}	◯	◯	◯	◯	◯
Abdominal pain {[abd_pain1] radio} {Branching logic (show if): [sick_today] = '1' or [sick_today] = '2'}	◯	◯	◯	◯	◯
Diarrhea {[diarrhea1] radio} {Branching logic (show if): [sick_today] = '1' or [sick_today] = '2'}	◯	◯	◯	◯	◯
Lost or changed of sense of taste {[loss_taste] radio} {Branching logic (show if): [sick_today] = '1' or [sick_today] = '2'}	◯	◯	◯	◯	◯
Lost or changed of sense of smell {[loss_smell] radio} {Branching logic (show if): [sick_today] = '1' or [sick_today] = '2'}	◯	◯	◯	◯	◯
Arm Soreness {[arm_sore] radio} {Branching logic (show if): [sick_today] = '1' or [sick_today] = '2'}	◯	◯	◯	◯	◯
Other {[oth1] radio} {Branching logic (show if): [sick_today] = '1' or [sick_today] = '2'}	◯	◯	◯	◯	◯

Temperature (F)
{[temp] text}
{Branching logic (show if): [fever] = '1' or [fever] = '2' or [fever] = '3' or [fever] = '4'}

____

Specify Other
{[oth_symp] text}
{Branching logic (show if): [oth1] = '1' or [oth1] = '2' or [oth1] = '3' or [oth1] = '4'}

____

COVID-19

Do you have any COVID-19 tests or results to report since your last survey response?<br>{[tested_corona_wkly] radio}	◯ {1} Yes ◯ {0} No<br>◯ {777} Don't Know
What type of test was it?<br>{[test_type_dly] radio}<br>{Branching logic (show if): [tested_corona_wkly] = '1'}	◯ {1} Antibody (via blood draw)<br>◯ {2} Diagnostic (PCR)<br>◯ {3} Antigen (rapid 15 minute test)<br>◯ {4} Don't know
What was the result?<br>{[reslt_testd_wkly] dropdown}<br>{Branching logic (show if): [tested_corona_wkly] = '1'}	◯ {1} Was not able to get tested<br>◯ {2} Positive ◯ {3} Negative<br>◯ {4} Still waiting for results<br>◯ {5} Inconclusive
Why?<br>{[not_able_covd_3] text}<br>{Branching logic (show if): [reslt_testd_wkly] = '1'}	______________________
Why?<br>{[not_able_covd_test] text}<br>{Branching logic (show if): [reslt_testd_wkly] = '1'}	______________________
Do you have a vaccine to report today or a vaccine that you haven't reported to us yet?<br>{[covid_daily_shot] radio}	◯ {1} Yes ◯ {0} No
Which COVID-19 vaccine did you receive?<br>{[covid_daily_shot_type] radio}<br>{Branching logic (show if): [covid_daily_shot] = '1'}	◯ {1} First: My vaccine requires two doses<br>◯ {2} First: My vaccine only requires one dose<br>◯ {3} Second dose<br>◯ {4} First Booster (or Third Dose)<br>◯ {5} Second Booster (or Fourth Dose)
What was the date of the dose?<br>{[covid_daily_shot_date] text date_mdy}<br>{Branching logic (show if): [covid_daily_shot] = '1'}	______________________
Do you know the brand?<br>{[covid_daily_shot_brand] radio}<br>{Branching logic (show if): [covid_daily_shot] = '1'}	◯ {1} Pfizer<br>◯ {2} Moderna<br>◯ {12} Johnson & Johnson<br>◯ {3} Other<br>◯ {11} I don't know

Did you have any symptoms that you believe were caused by the dose?

	{0} None	{1} Mild	{2} Moderate	{3} Severe	{4} Very Severe
Fever<br>{[fever_daily_shot] radio}<br>{Branching logic (show if): [covid_daily_shot] = '1'}	◯	◯	◯	◯	◯

Generally feeling ill (Malaise) {[malaise_daily_shot] radio} {Branching logic (show if): [covid_daily_shot] = '1'}	○	○	○	○	○
Cough {[cough1_daily_shot] radio} {Branching logic (show if): [covid_daily_shot] = '1'}	○	○	○	○	○
Sneezing {[sneezing_daily_shot] radio} {Branching logic (show if): [covid_daily_shot] = '1'}	○	○	○	○	○
Shortness of breath/difficulty breathing {[shortness1_daily_shot] radio} {Branching logic (show if): [covid_daily_shot] = '1'}	○	○	○	○	○
Muscle Aches {[muscle_aches_daily_shot] radio} {Branching logic (show if): [covid_daily_shot] = '1'}	○	○	○	○	○
Runny/stuffy nose {[runny_nose_daily_shot] radio} {Branching logic (show if): [covid_daily_shot] = '1'}	○	○	○	○	○
Throat discomfort (sore/scratchy throat) {[sore_throat_daily_shot] radio} {Branching logic (show if): [covid_daily_shot] = '1'}	○	○	○	○	○
Nausea or vomiting {[nausea1_daily_shot] radio} {Branching logic (show if): [covid_daily_shot] = '1'}	○	○	○	○	○
Headache {[headache1_daily_shot] radio} {Branching logic (show if): [covid_daily_shot] = '1'}	○	○	○	○	○
Abdominal pain {[abd_pain1_daily_shot] radio} {Branching logic (show if): [covid_daily_shot] = '1'}	○	○	○	○	○
Diarrhea {[diarrhea1_daily_shot] radio} {Branching logic (show if): [covid_daily_shot] = '1'}	○	○	○	○	○

Lost or changed of sense of taste

{[loss_taste_daily_shot] radio}
{Branching logic (show if):
[covid_daily_shot] = '1'}

Lost or changed of sense of smell
{[loss_smell_daily_shot] radio}
{Branching logic (show if):
[covid_daily_shot] = '1'}

Arm Soreness
{[arm_sore_daily_shot] radio}
{Branching logic (show if):
[covid_daily_shot] = '1'}

Other
{[oth1_daily_shot] radio}
{Branching logic (show if):
[covid_daily_shot] = '1'}

Have you received any treatment for COVID-19 since your last survey?
{[covid_treatment] radio}

◯ {1} Yes ◯ {0} No

Please describe what treatment you received:
{[covid_treatment_desc] text}
{Branching logic (show if): [covid_treatment] = '1'}

____

Flu

Were you tested for the flu since your last survey?
{[tested_flu_today] radio}
{Branching logic (show if): [sick_today] = '1'}

◯ {1} Yes ◯ {0} No
◯ {777} Don't Know

What was the result?
{[reslt_testd_wkly_2] dropdown}
{Branching logic (show if): [tested_flu_today] = '1'}

◯ {1} Was not able to get tested
◯ {2} Positive ◯ {3} Negative
◯ {4} Still waiting for results
◯ {5} Inconclusive

Why?
{[not_able_covd_2] text}
{Branching logic (show if): [reslt_testd_wkly_2] = '1'}

____

Why?
{[not_able_flu] text}
{Branching logic (show if): [reslt_testd_wkly_2] = '1'}

____

Did you receive any other diagnosis to explain your symptoms?
{[oth_diagnosis] yesno}
{Branching logic (show if): [sick_today] = '1'}

◯ Yes ◯ No

What was it?
{[spef_oth_diagnosis] text}
{Branching logic (show if): [oth_diagnosis] = '1'}

____

Did you take any new medications within the past 24 hours?
{[medications_past24] yesno}
{Branching logic (show if): [sick_today]='1'}

◯ Yes ◯ No

What medications?
{[medications_past24_list] textarea}
{Branching logic (show if): [medications_past24]='1'}

____

Hospital Admission(s)

Were you admitted to the hospital since your last survey response?
{[admtd_hosp] yesno}
{Branching logic (show if): [sick_today] = '1'}

◯ Yes ◯ No

What was the date you were admitted?
{[date_testd_wkly_3] text date_mdy}
{Branching logic (show if): [admtd_hosp] = '1'}

____

Do you want to opt out and withdraw from the study?

{[pre_optout_daily] yesno}

◯ Yes ◯ No

Your participation in this study is very helpful as we learn about COVID-19. However, if you would like to 'opt-out' of this study, please click here
{[optout_daily] radio}
{Branching logic (show if): [pre_optout_daily]='1'}

◯ {1} Opt-out

Opt-Out (If greater that 1, then participant has opted out)
{[optout_sum_2] calc}

____

Drp Tools

Email Project ID
{[email_proj_id] text} ______________________________

Date Calculated Fields

Day #1<br>{[day1] text date_mdy}	______________________
Day #2<br>{[day2] text date_mdy}	______________________
Day #3<br>{[day3] text date_mdy}	______________________
Day #4<br>{[day4] text date_mdy}	______________________
Day #6<br>{[day6] text date_mdy}	______________________
Day #5<br>{[day5] text date_mdy}	______________________
Day #7<br>{[day7] text date_mdy}	______________________
Day #8<br>{[day8] text date_mdy}	______________________
Day #9<br>{[day9] text date_mdy}	______________________
Day #10<br>{[day10] text date_mdy}	______________________
Day #11<br>{[day11] text date_mdy}	______________________
Day #12<br>{[day12] text date_mdy}	______________________
Day #13<br>{[day13] text date_mdy}	______________________
Day #14<br>{[day14] text date_mdy}	______________________
Day #15<br>{[day15] text date_mdy}	______________________
Day #16<br>{[day16] text date_mdy}	______________________
Day #17<br>{[day17] text date_mdy}	______________________

Day #18
{[day18] text date_mdy} ________________________________

Day #19
{[day19] text date_mdy} ________________________________

Day #20
{[day20] text date_mdy} ________________________________

Day #21
{[day21] text date_mdy} ________________________________

Day #22
{[day22] text date_mdy} ________________________________

Day #23
{[day23] text date_mdy} ________________________________

Day #24
{[day24] text date_mdy} ________________________________

Day #25
{[day25] text date_mdy} ________________________________

Day #26
{[day26] text date_mdy} ________________________________

Day #27
{[day27] text date_mdy} ________________________________

Day #28
{[day28] text date_mdy} ________________________________

Day #29
{[day29] text date_mdy} ________________________________

Day #30
{[day30] text date_mdy} ________________________________

Day #31
{[day31] text date_mdy} ________________________________

Day #32
{[day32] text date_mdy} ________________________________

Day #33
{[day33] text date_mdy} ________________________________

Day #34
{[day34] text date_mdy} ________________________________

Day #35
{[day35] text date_mdy} ________________________________

Day #36
{[day36] text date_mdy} ______________________________

Day #37
{[day37] text date_mdy} ______________________________

Day #38
{[day38] text date_mdy} ______________________________
