# Supplementary material for: A Method for Intelligent Allocation of Diagnostic Testing by Leveraging Data from Commercial Wearable Devices: A Case Study on COVID-19
Source: Res Sq. 2022 Apr 1:rs.3.rs-1490524. Preprint. [Version 1] doi: 10.21203/rs.3.rs-1490524/v1 (PMC8978951; doi:10.21203/rs.3.rs-1490524/v1)
Supplement: Supplement 2 [file de6c43bf95fef8b26ea951e6.docx]

**Supplementary Fig 1**

**
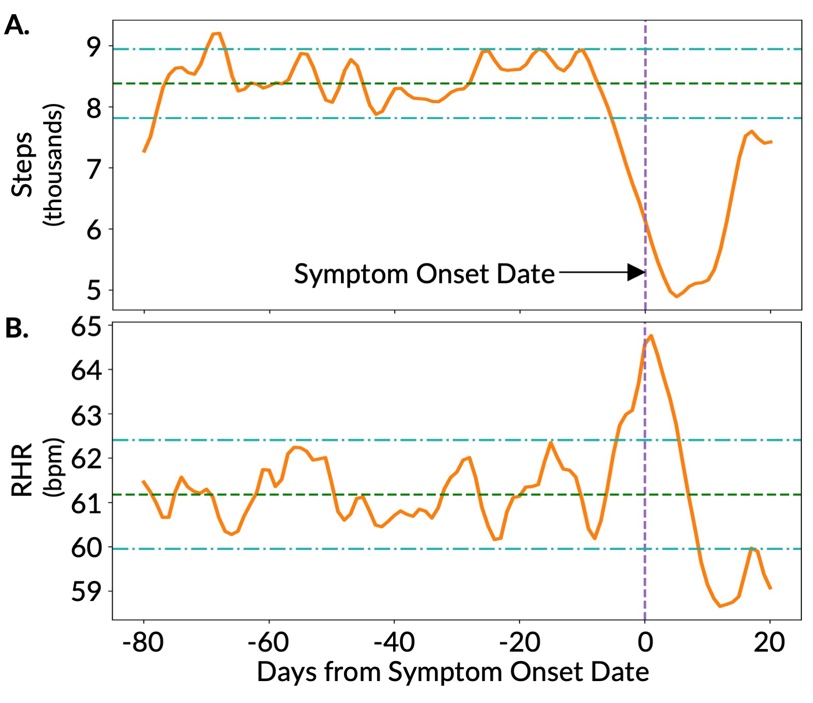
**

**Supplementary Fig. 1. Time-series plot of digital biomarkers with respect to symptom onset date.** (A) Time-averaged step count and (B) time-averaged resting heart rate (RHR) of the subset of participants (N=33) in the training set with available symptom onset date who tested positive for COVID-19. The horizontal green dashed lines and the horizontal light green dash-dotted lines show the baseline period mean and ± 2 standard deviations from the baseline mean respectively.

**Supplementary Fig 2**

**
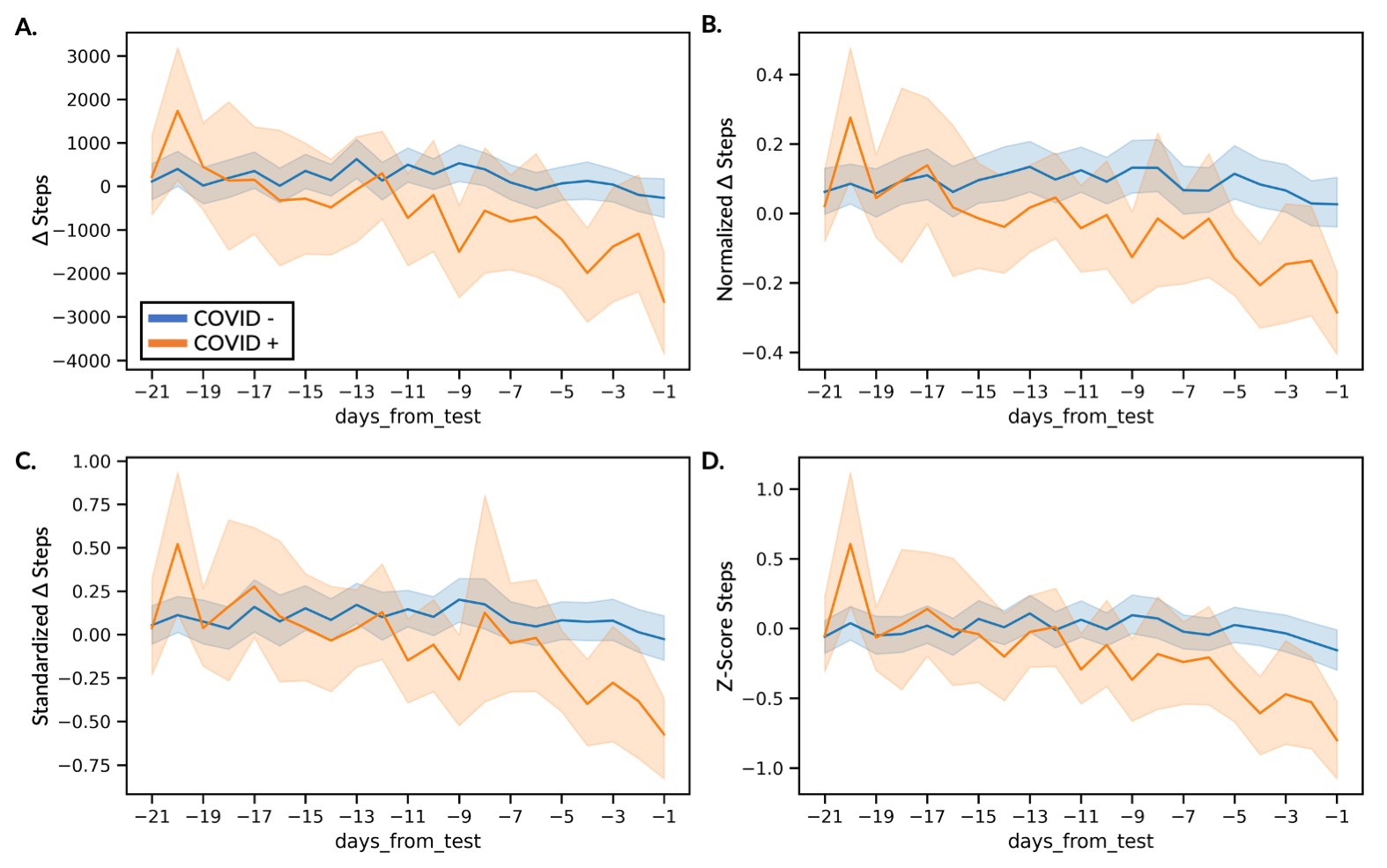
**

**Supplementary Fig. 2. Time-series plot of four deviation metrics from the steps digital biomarker for the AF cohort.** (A) deviation in step count from baseline median (detection – baseline median), ΔSteps, (B) normalized ΔSteps, (C) standardized ΔSteps, and (D) z-score ((detection – baseline mean) / baseline standard deviation) of Steps between the participants diagnosed as COVID-19 positive and negative. Confidence bands show the 95% confidence intervals.

**Supplementary Fig 3**

**
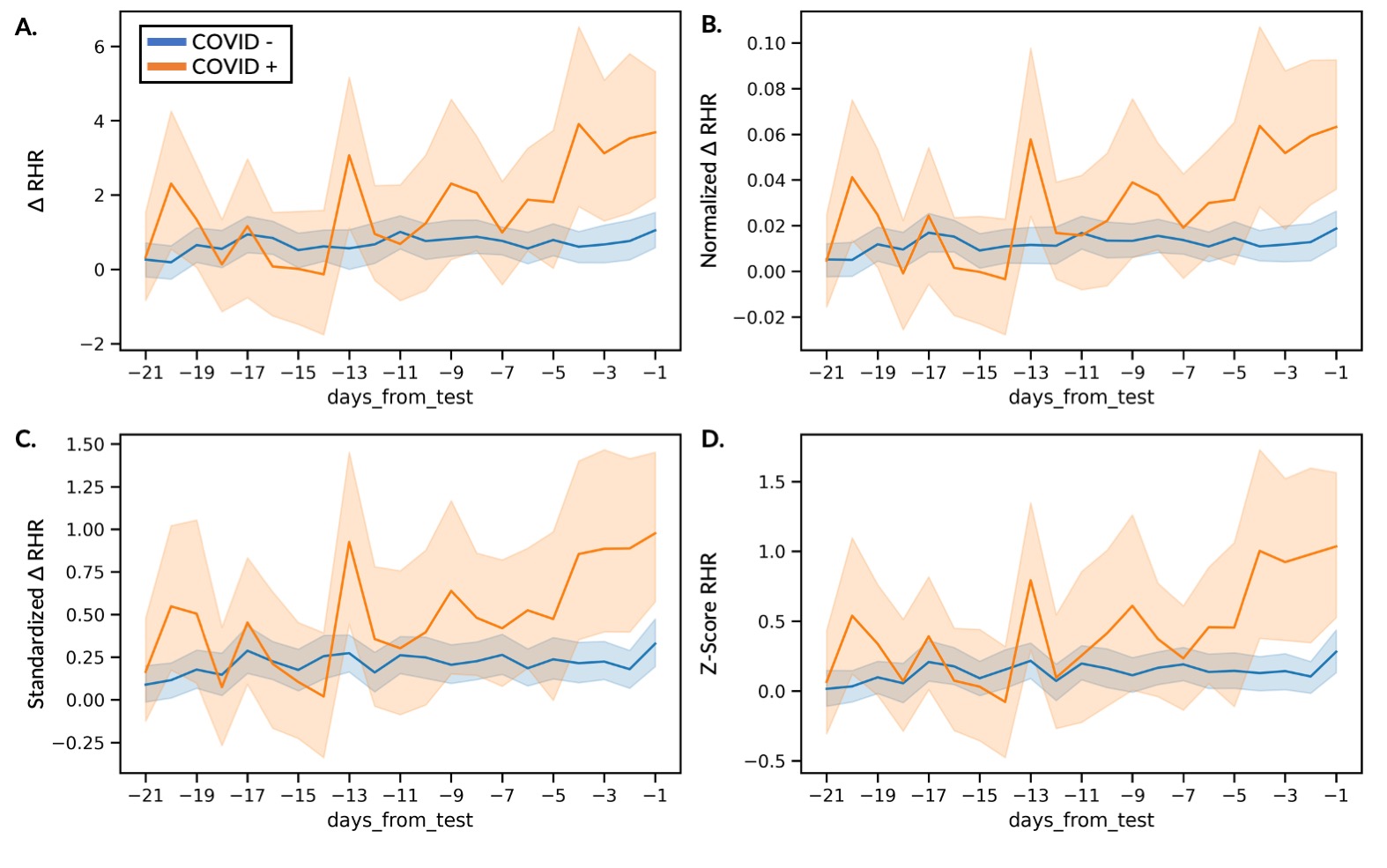
**

**Supplementary Fig. 3. Time-series plot of four deviation metrics from the RHR digital biomarker for the AF cohort.** (A) deviation in RHR from baseline median (detection – baseline median), ΔRHR, (B) normalized ΔRHR, (C) standardized ΔRHR, and (D) z-score ((detection − baseline mean) / baseline standard deviation) of RHR between the participants diagnosed as COVID-19 positive and negative. Confidence bands are showing the 95% confidence intervals.

**Supplementary Fig 4**

**
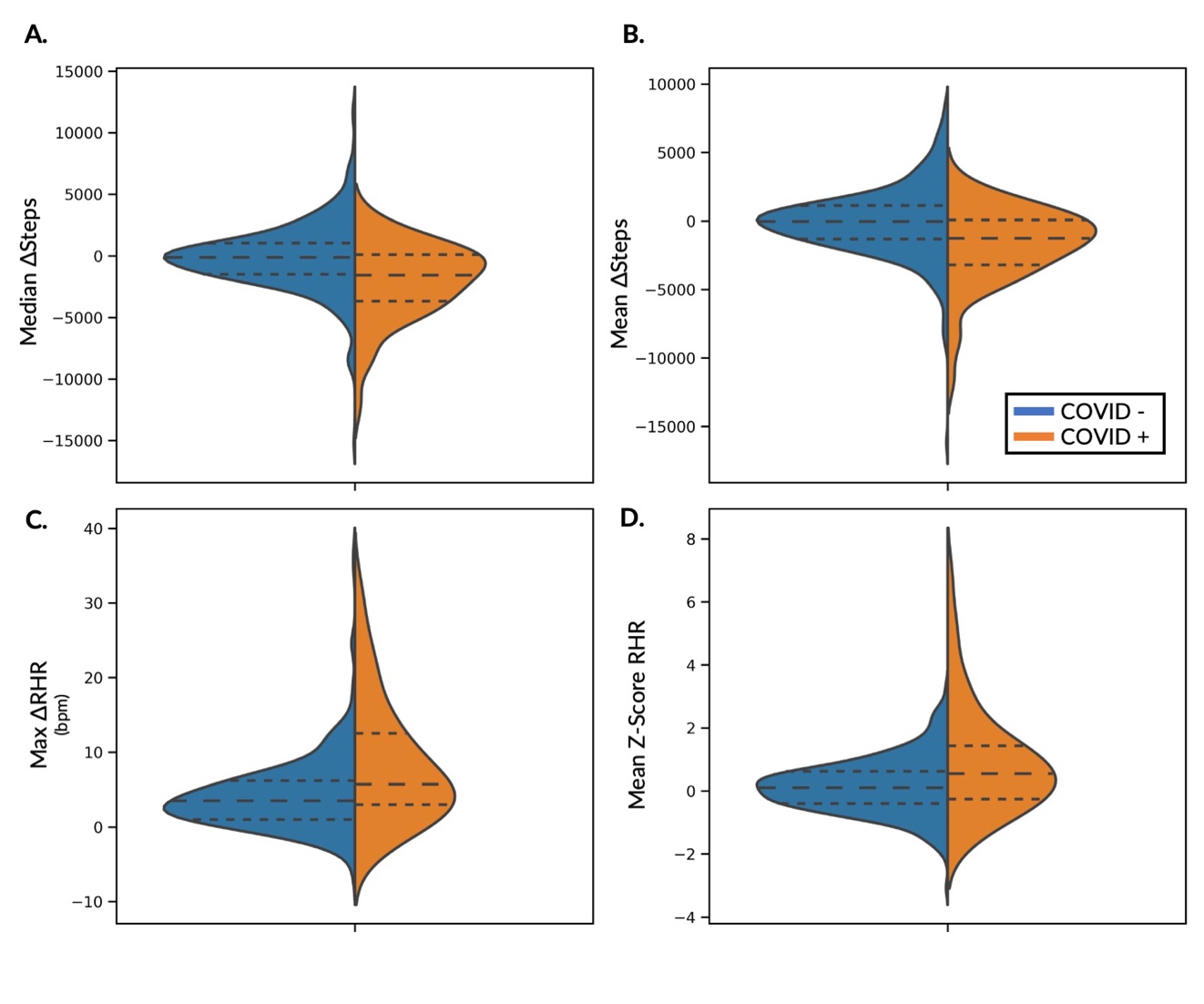
**

**Supplementary Fig. 4. Distribution of the top two steps and RHR features across COVID-19 positive and negative groups in the AF cohort.** Violin plots of two steps **(A, B)** and RHR **(C, D)** features with the lowest p-values, showing the distribution of the features between the participants diagnosed as COVID-19 positive (orange) and negative (blue). The outer dashed lines represent the first and third quartiles and the inner dashed line represents the median.

**Supplementary Fig 5**

**
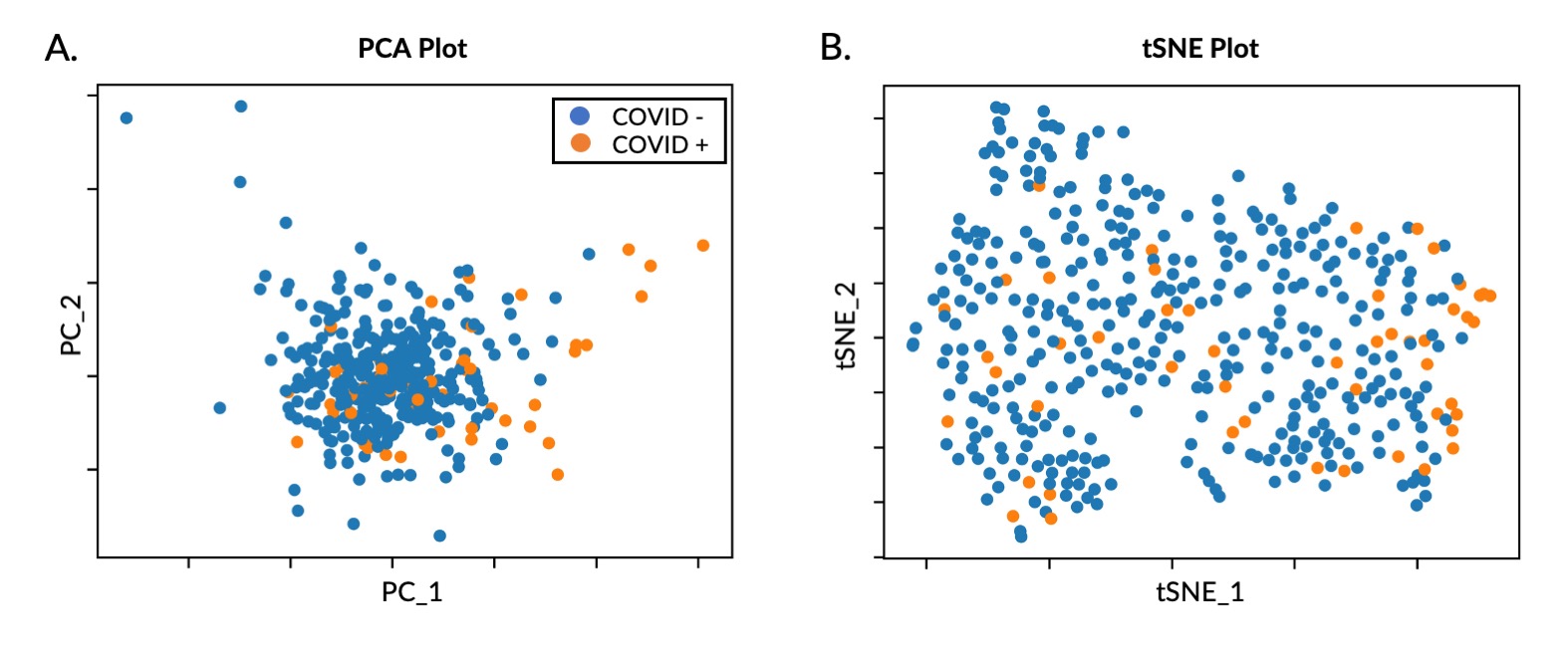
**

**Supplementary Fig. 5. Low dimensional representation of input variables for the ITA model using the AF cohort.** **(A)** Principal component analysis (PCA) and **(B)** t-stochastic neighborhood embedding (tSNE) plots for the input variables (significant steps and RHR features).

**Supplementary Table 1: Demographic information for participants in the CovIdentify study**


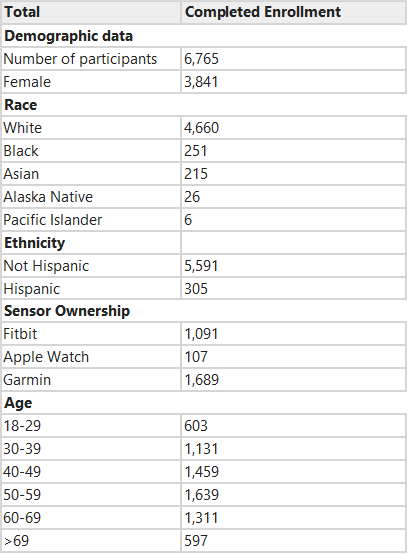


**Supplementary Table 2: Significant features across three cohorts ordered in an ascending order based on significance (p-value).**

| **AF Cohort** | **AHF Cohort** | **FHF Cohort** |
| --- | --- | --- |
| Mean Z-score RHR | Median Z-score RHR | Max ΔRHR |
| Max ΔRHR | Mean Z-score RHR | Max Normalized ΔRHR |
| Max Normalized ΔRHR | Max Z-score RHR | Range ΔRHR |
| Median Z-score RHR | Median Standardized ΔRHR | Range Normalized ΔRHR |
| Max Z-score RHR | Mean Standardized ΔRHR | Max Z-score RHR |
| Mean Normalized ΔRHR | Max Normalized ΔRHR | Mean Normalized ΔRHR |
| Mean Standardized ΔRHR | Max ΔRHR | Mean ΔRHR |
| Median Normalized ΔRHR | Mean ΔSteps | Max Standardized ΔRHR |
| Mean Standardized ΔRHR | Median ΔSteps | Mean Z-score RHR |
| Median ΔSteps | Mean Normalized ΔRHR | Mean ΔSteps |
| Mean ΔRHR | Median Normalized ΔRHR | Median ΔSteps |
| Max Standardized ΔRHR | Mean ΔRHR | Median Normalized ΔRHR |
| Median ΔRHR | Max Standardized ΔRHR | Median ΔRHR |
| Mean ΔSteps | Median ΔRHR | Median Z-score Steps |
| Mean Z-score Steps | Mean Z-score Steps | Mean Z-score Steps |
| Median Z-score Steps | Median Z-score Steps | Median Z-score RHR |
| Mean Normalized ΔSteps | Max Z-score Steps | Mean Standardized ΔRHR |
| Mean Standardized ΔSteps | Mean Normalized ΔSteps | Range Z-score RHR |
| Median Standardized ΔSteps | Median Standardized ΔSteps | Median Standardized ΔSteps |
| Max Z-score Steps | Mean Standardized ΔSteps | Mean Standardized ΔSteps |
| Median Normalized ΔSteps | Max ΔSteps | Median Standardized ΔRHR |
| Min ΔSteps | Min ΔSteps | Min ΔSteps |
| Max Standardized ΔSteps | Median Normalized ΔSteps | Max Z-score Steps |
| Max Normalized ΔSteps | Max Normalized ΔSteps | Mean Normalized ΔSteps |
| Min Normalized ΔSteps | Max Standardized ΔSteps | Max ΔSteps |
| Max ΔSteps | Min Z-score RHR | Max Standardized ΔSteps |
| Min Z-score RHR | Min Standardized ΔRHR | Median Normalized ΔSteps |
| Min Standardized ΔRHR | Min Normalized ΔSteps | Min Normalized ΔSteps |
| Min Z-score Steps |  | Max Normalized ΔSteps |
|  |  | Min Z-score Steps |
|  |  | Min Z-score RHR |

Max, Maximum; Min, Minimum.
