# Supplementary material for: A Method for Intelligent Allocation of Diagnostic Testing by Leveraging Data from Commercial Wearable Devices: A Case Study on COVID-19
Source: Res Sq. 2022 Apr 1:rs.3.rs-1490524. Preprint. [Version 1] doi: 10.21203/rs.3.rs-1490524/v1 (PMC8978951; doi:10.21203/rs.3.rs-1490524/v1)
Supplement: Supplement 3 [file 02fa26976804fc8f7c042f47.docx]

**Extended Data Fig 1**

**
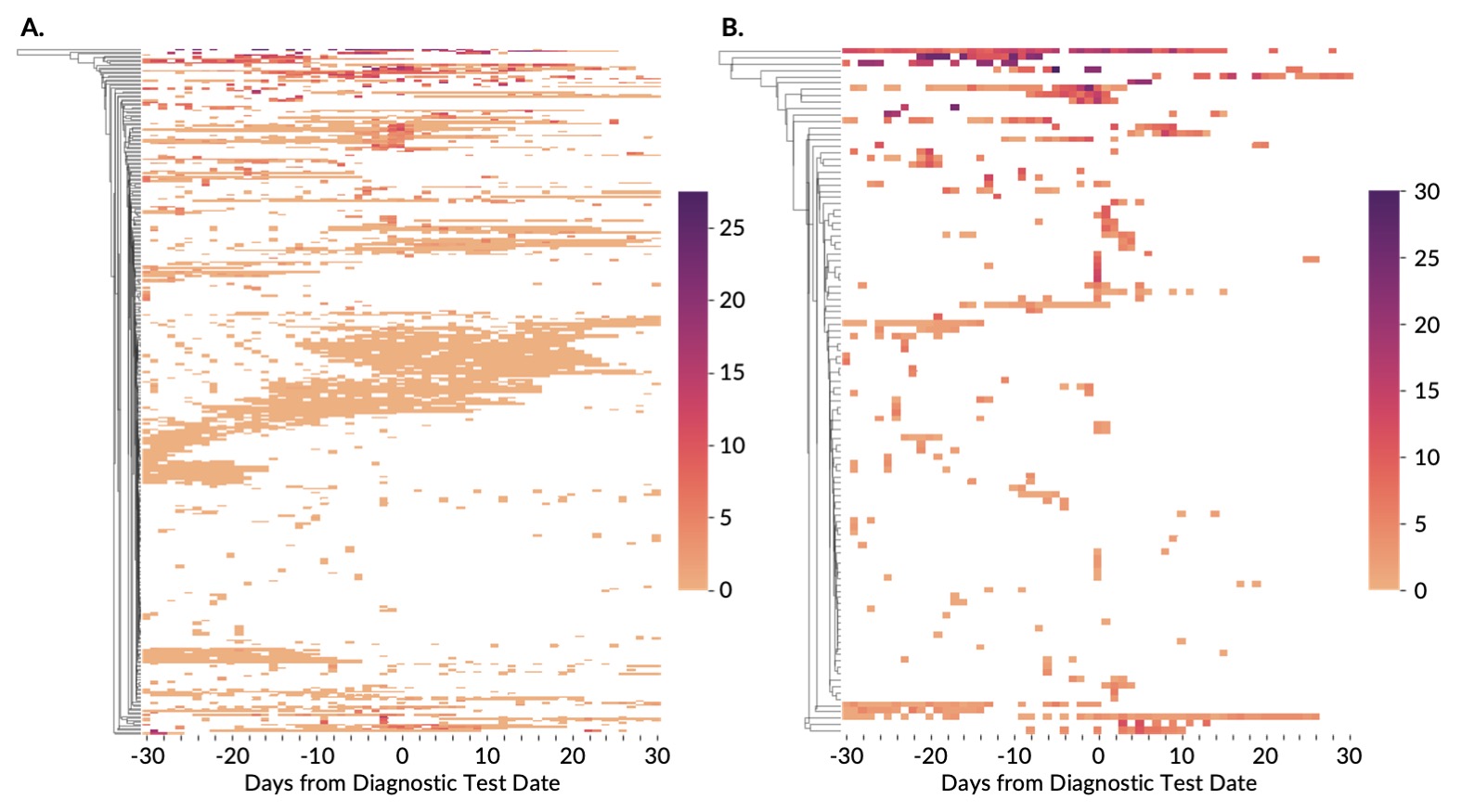
**

**Extended Data Fig. 1. Hierarchical clustering of COVID-19 negative participants.**

**A.** CovIdentify participants (n = 348) **B.** MyPHD participants (n =108). Each participant is represented by a row on the heatmap to the right of the dendrogram. The color scale demonstrates the daily sum of the symptom ratings, where each symptom ranged on a scale from 1 to 5 (purple = most symptomatic and peach = least symptomatic). Areas in white show where no symptoms were reported.

**Extended Data Fig 2**

**
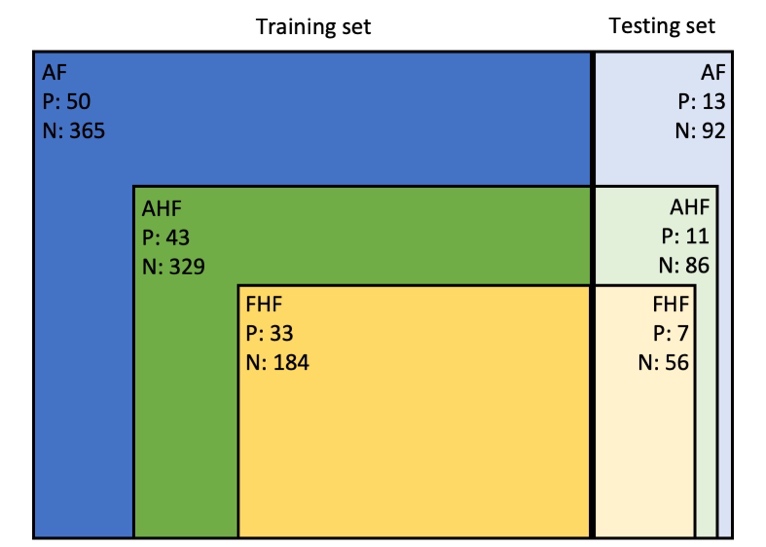
**

**Extended Data Fig. 2. Cohort definitions.** All three cohorts (All Frequency, AF (blue), All-High-Frequency, AHF (green), and Fitbit-High-Frequency, FHF (yellow)) were divided into an 80% train and 20% test split, with FHF as a subgroup of AHF, which itself is a subset of AF to ensure that no observations in the training set of one cohort existed in the test set of another.

**Extended Data Fig 3**

**
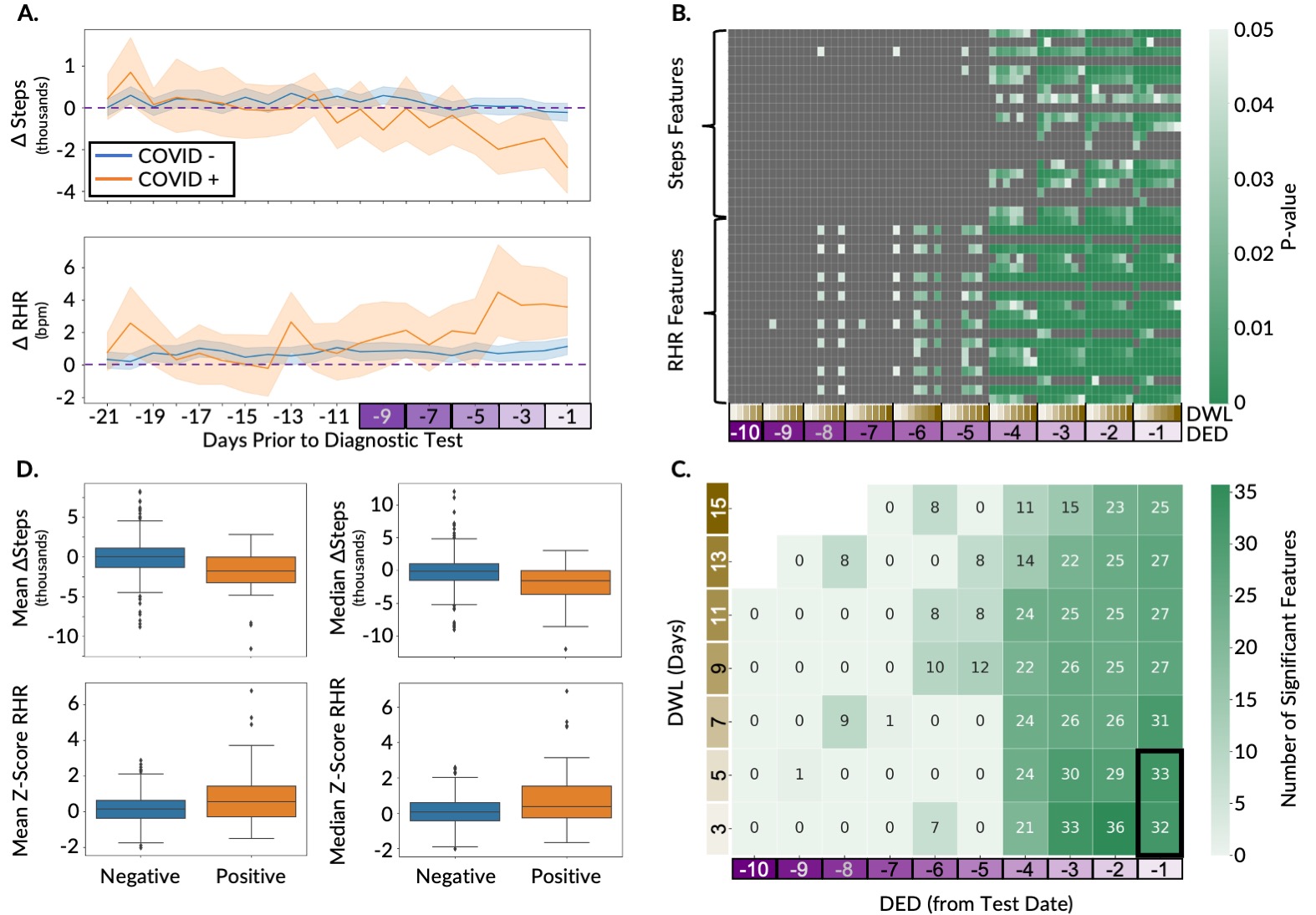
**

**Extended Data Fig. 3. Overview of the feature exploration and extraction for the ITA model development on the AHF cohort. A.** Time-series plot of the deviation in digital biomarkers (ΔSteps and ΔRHR) in the detection window compared to baseline periods, between the participants diagnosed as COVID-19 positive and negative. The horizontal dashed line displays the baseline median and the confidence bounds show the 95% confidence intervals. **B.** Heatmaps of steps and RHR features that are statistically significantly different (p-value < 0.05) in a grid search with different DED and DWL combinations, with green boxes showing p-values < 0.05 and gray boxes showing p-values ≥ 0.05. The p-values are adjusted with the Benjamini-Hochberg method for multiple hypothesis correction. **C.** Summary of the significant features (p-value < 0.05) from B, with each box showing the number of statistically significant features for the different combinations of DED and DWL. The intersection of the significant features across DWL of 3 and 5 days with a common DED of 1 day prior to the test date (as shown using the black rectangle) were used for the ITA model development. **D.** Box plots comparing the distribution of the two most significant steps and RHR features between the participants diagnosed as COVID-19 positive and negative.

**Extended Data Fig 4**

**
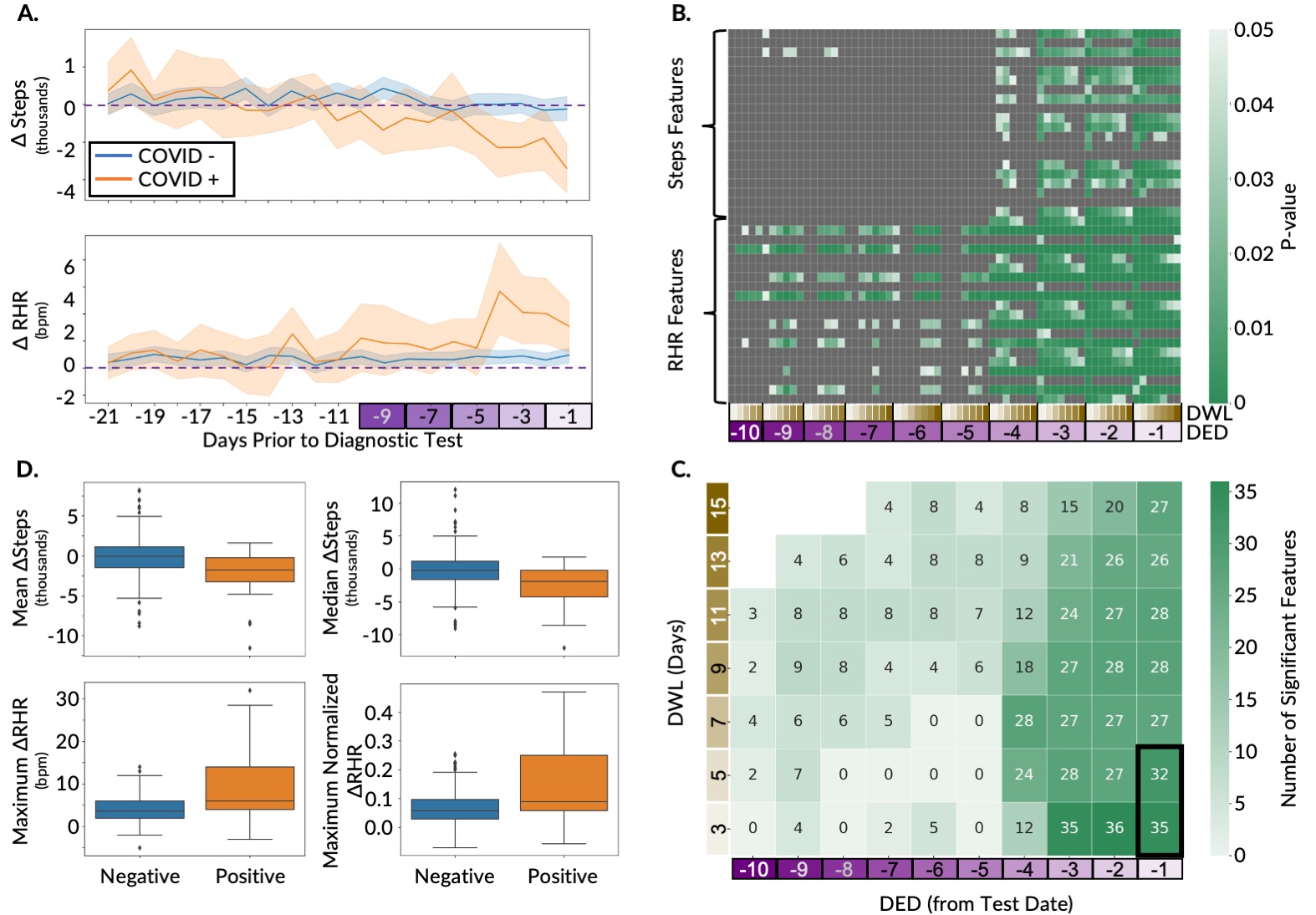
**

**Extended Data Fig. 4. Overview of the feature exploration and extraction for the ITA model development on the FHF cohort. A.** Time-series plot of the deviation in digital biomarkers (ΔSteps and ΔRHR) in the detection window compared to baseline periods, between the participants diagnosed as COVID-19 positive and negative. The horizontal dashed line displays the baseline median and the confidence bounds show the 95% confidence intervals. **B.** Heatmaps of steps and RHR features that are statistically significantly different (p-value < 0.05) in a grid search with different DED and DWL combinations, with green boxes showing p-values < 0.05 and gray boxes showing p-values ≥ 0.05. The p-values are adjusted with the Benjamini-Hochberg method for multiple hypothesis correction. **C.** Summary of the significant features (p-value < 0.05) from B, with each box showing the number of statistically significant features for the different combinations of DED and DWL. The intersection of the significant features across DWL of 3 and 5 days with a common DED of 1 day prior to the test date (as shown using the black rectangle) were used for the ITA model development. **D.** Box plots comparing the distribution of the two most significant steps and RHR features between the participants diagnosed as COVID-19 positive and negative.

**Extended Data Fig 5**

**
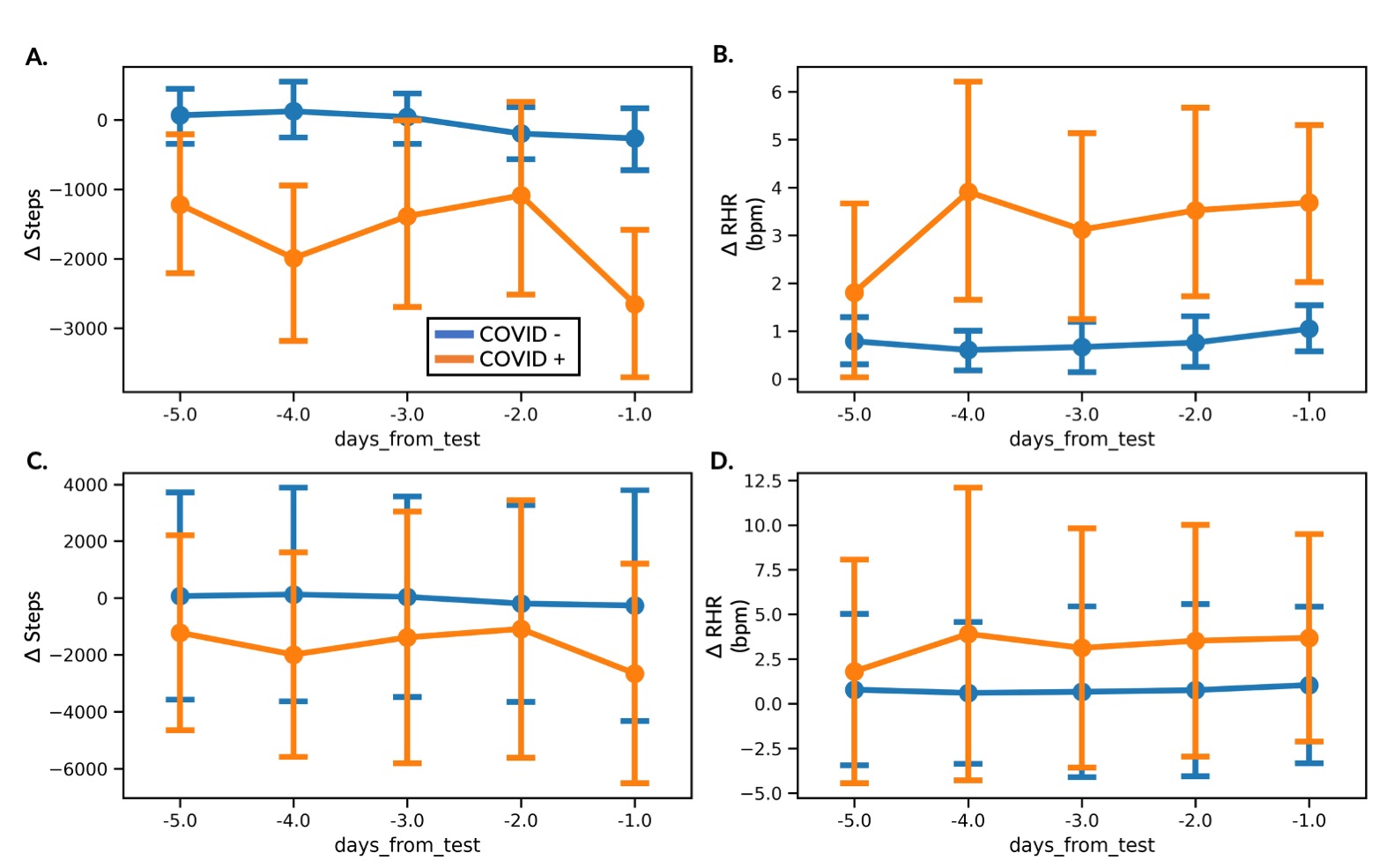
**

**Extended Data Fig. 5. Overview of the digital biomarkers in the detection period showing different representations of error bars.** Time-series plot of Δ Steps **(A, C)**, and Δ RHR **(B, D)** between the participants diagnosed as COVID-19 positive (orange) and negative (blue). Error bars in A and B show the 95% confidence intervals, whereas error bars in C and D show the one standard deviation.

**Extended Data Fig 6**

**
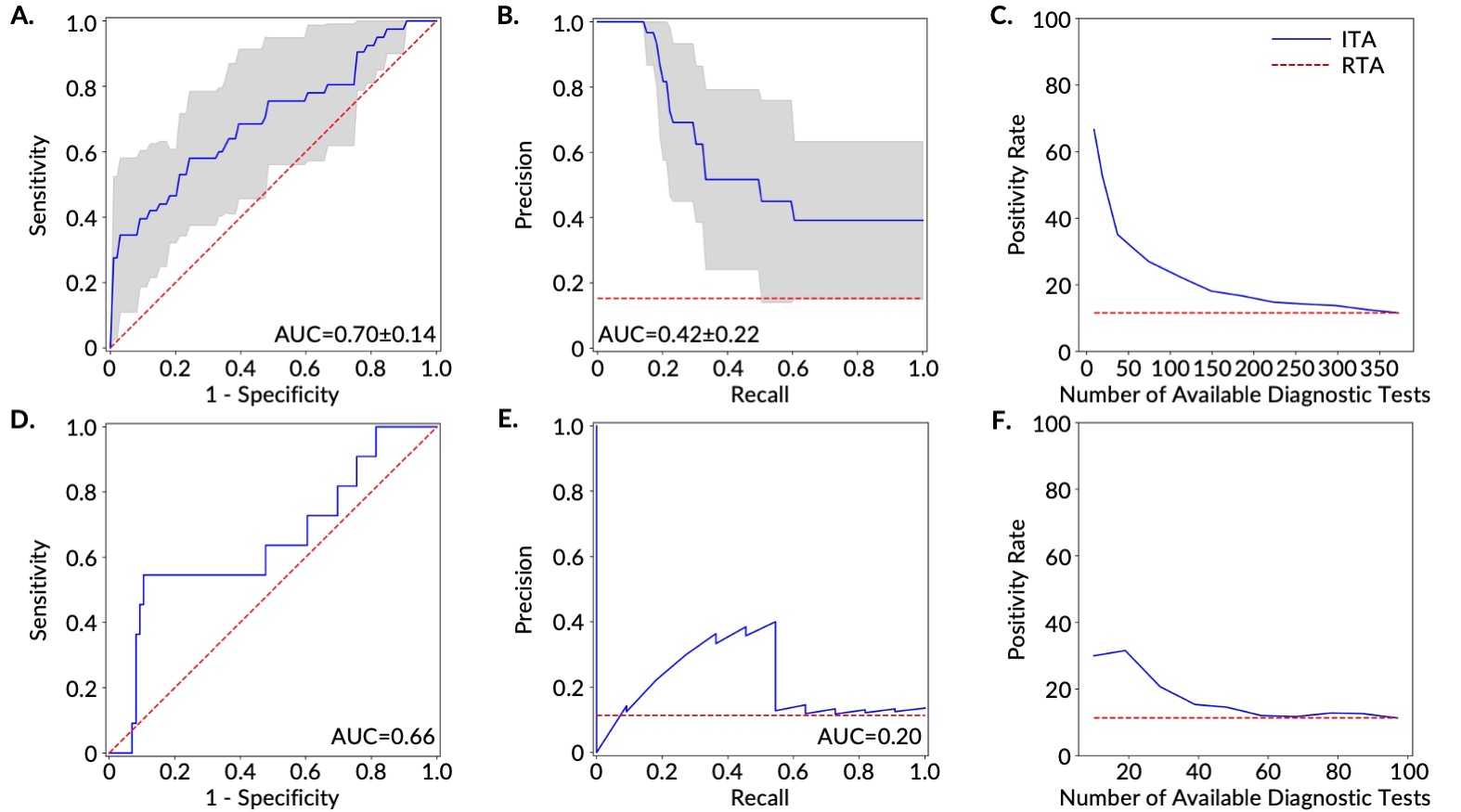
**

**Extended Data Fig. 6. Prediction and ranking results of the ITA models on both the training (A-C) and testing sets (D-F) for the AHF Cohort. A.** Receiver operating characteristics curves (ROCs) and **B.** precision-recall curves (PRCs) for the discrimination between COVID-19 positive participants (n=43) and negative participants (n=329) in the training set. The gray area shows one standard deviation from the mean of the ROCs/PRCs generated from 10-fold nested cross-validation on the training set and the red dashed line shows the results based on a Random Testing Allocation (RTA) model (the null model). **C.** The positivity rate of the diagnostic testing subpopulation as determined by ITA given a specific number of available diagnostic tests. The red dashed line displays the positivity rate/pre-test probability of an RTA (null) model. **D.** ROC and **E.** PRC for the discrimination between Covid-19 positive participants (n=11) and negative participants (n=86) in the test set. The red dashed line shows the results based on an RTA model. **F.** Positivity rate of the diagnostic testing subpopulation as determined by ITA given a specific number of available diagnostic tests. The red dashed line shows the positivity rate of an RTA (null) model.

**Extended Data Fig 7**

**
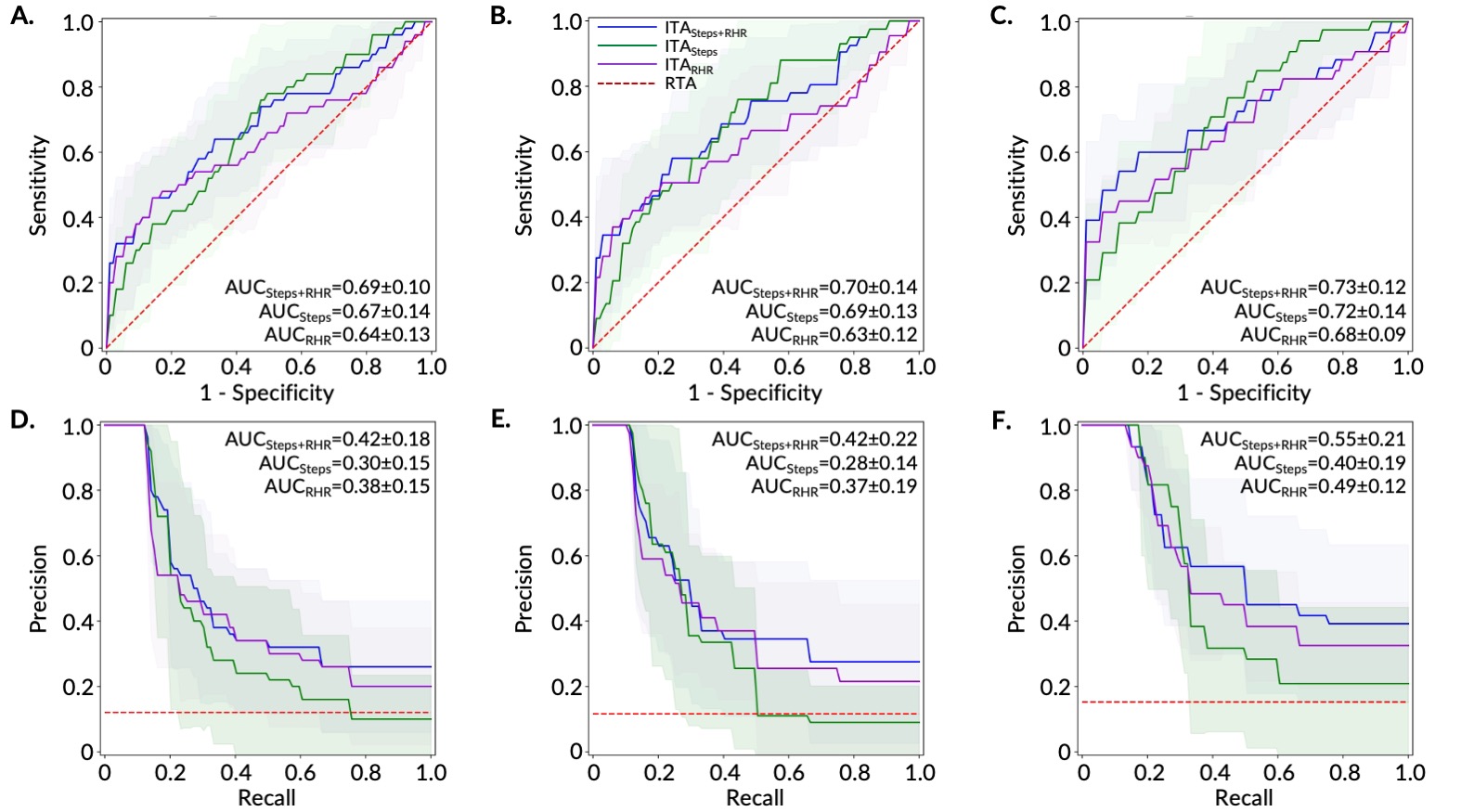
**

**Extended Data Fig. 7. Comparison of model performance on all three cohorts using features from combination of Steps and RHR, Steps and RHR digital biomarkers.** **(A, B, C)** ROC curves and **(D, E, F)** PR curves generated from the model built on the training set of the three cohorts (AF, AHF, and FHF) and evaluated with 10-fold nested cross-validation. The light blue, light green, and light violet area show one standard deviation from the mean of the ROCs/PRs generated from 10-fold nested cross-validation on the training set for the models with Steps+RHR, Steps, and RHR features, respectively. The red dashed line shows the results based on a random testing allocation (RTA), or null model.

**Extended Data Fig 8**

**
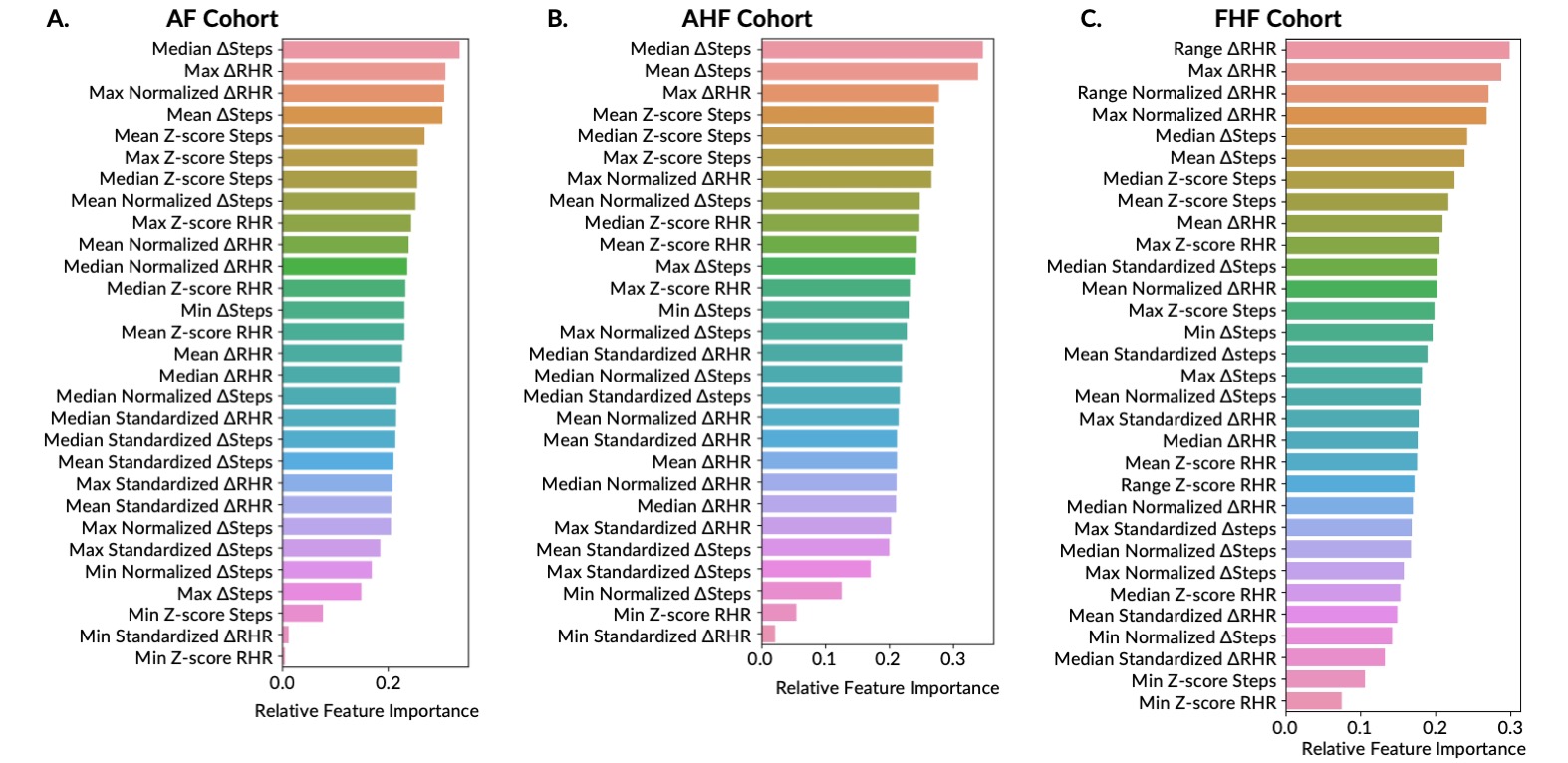
**

**Extended Data Fig. 8. Feature importance ranking for the logistic regression models.** Feature importance ranking based on the absolute value of coefficients of each feature used in the logistic regression model using training data from the **(A)** AF **(B)** AHF, and **(C)** FHF cohorts. Max, maximum; Min, minimum.

**Extended Data Table 1: Performance of the five machine learning algorithms tested for the ITA model.**

| **Cohort** | **AF** | | **AHF** | | **FHF** | |
| --- | --- | --- | --- | --- | --- | --- |
| **Model** | **AUC-ROC** | **AUC-PR** | **AUC-ROC** | **AUC-PR** | **AUC-ROC** | **AUC-PR** |
| **Logistic Regression** | 0.69±0.10 | 0.42±0.18 | 0.70±0.14 | 0.42±0.22 | 0.73±0.12 | 0.55±0.21 |
| **KNN** | 0.61±0.11 | 0.32±0.17 | 0.61±0.11 | 0.32±0.18 | 0.67±0.12 | 0.48±0.21 |
| **SVM** | 0.63±0.12 | 0.27±0.17 | 0.64±0.13 | 0.32±0.20 | 0.64±0.16 | 0.45±0.16 |
| **Random Forest** | 0.67±0.13 | 0.38±0.17 | 0.68±0.15 | 0.41±0.20 | 0.71±0.14 | 0.5±0.16 |
| **XGBoost** | 0.67±0.14 | 0.35±0.19 | 0.71±0.12 | 0.4±0.20 | 0.71±0.13 | 0.45±0.20 |

Results reported in this table are generated from the model developed on the training set of the three cohorts using 10-fold nested cross-validation. The cross-validation results are calculated based on the validation on the left-out-fold in each iteration of the outer loop of the nested cross-validation and are reported in the mean (± SD) format.

**Extended Data Table 2: ITA model performance for symptomatic and asymptomatic individuals.**

| **Cohorts** | **Total Dataset** | | | | **ITA Determined Subpopulation**  **(30% Testing Capacity)** | | | |
| --- | --- | --- | --- | --- | --- | --- | --- | --- |
|  | **Total N** | **Total COVID+** | **Total COVID+**  **Symptomatic** | **Total**  **COVID+**  **Asymptomatic** | **Total N** | **Total COVID+** | **Total COVID+**  **Symptomatic** | **Total**  **COVID+**  **Asymptomatic** |
| **Training Set** | | | | | | | | |
| **AF** | 415 | 50 | 29 | 21 | 124 | 26 | 19 | 7 |
| **AHF** | 372 | 43 | 29 | 14 | 112 | 25 | 20 | 5 |
| **FHF** | 217 | 33 | 21 | 12 | 65 | 20 | 16 | 4 |
| **Test Set** | | | | | | | | |
| **AF** | 105 | 13 | 8 | 5 | 32 | 6 | 5 | 1 |
| **AHF** | 97 | 11 | 8 | 3 | 29 | 6 | 5 | 1 |
| **FHF** | 63 | 7 | 6 | 1 | 19 | 5 | 4 | 1 |
